# Mixture Complexity and Its Application to Gradual Clustering Change Detection

**DOI:** 10.3390/e24101407

**Published:** 2022-10-01

**Authors:** Shunki Kyoya, Kenji Yamanishi

**Affiliations:** Graduate School of Information Science and Technology, The University of Tokyo, 7-3-1 Hongo, Bunkyo-ku, Tokyo 113-8656, Japan

**Keywords:** finite mixture model, clustering, change detection, gradual change, information theory

## Abstract

We consider measuring the number of clusters (cluster size) in the finite mixture models for interpreting their structures. Many existing information criteria have been applied for this issue by regarding it as the same as the number of mixture components (mixture size); however, this may not be valid in the presence of overlaps or weight biases. In this study, we argue that the cluster size should be measured as a continuous value and propose a new criterion called mixture complexity (MC) to formulate it. It is formally defined from the viewpoint of information theory and can be seen as a natural extension of the cluster size considering overlap and weight bias. Subsequently, we apply MC to the issue of gradual clustering change detection. Conventionally, clustering changes have been regarded as abrupt, induced by the changes in the mixture size or cluster size. Meanwhile, we consider the clustering changes to be gradual in terms of MC; it has the benefits of finding the changes earlier and discerning the significant and insignificant changes. We further demonstrate that the MC can be decomposed according to the hierarchical structures of the mixture models; it helps us to analyze the detail of substructures.

## 1. Introduction

### 1.1. Motivation

Finite mixture models are widely used for model-based clustering (for overviews and references see McLachlan and Peel [1] and Fraley and Raftery [2]). In this field, determining the number of components is a typical issue. It refers to the following two aspects: the number of elements used to represent the density distribution and the number of clusters used to group the data (referred to as *mixture size* and *cluster size*, respectively). In this study, we consider the problem of interpreting the cluster size when the mixture size is given. Many existing information criteria have been applied for this issue by regarding it as the same as mixture size; however, it may not be valid when the components have overlaps or weight biases. Therefore, we need to reconsider the definitions and meanings of the cluster size.

**Figure 1:**
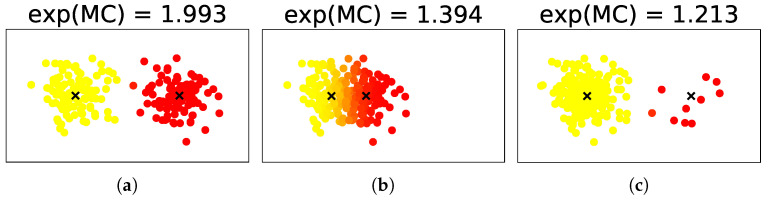
Examples of MC with Gaussian mixture models with a mixture size of two.

For instance, let us observe three cases of the Gaussian mixture model, as shown in Figure 1. Although the mixture size is two in any case, the situations are different. In case (a), the two components are distinct from each other and their weights are not biased; therefore, it is sound to believe that the cluster size is two as well. Meanwhile, in case (b), although their weights are not biased, the two components are very close to each other; then, as proposed in the work of Hennig [3], we may need to regard them as one cluster by merging them. In case (c), although the two components are distinct from each other, their weights are biased; as proposed in Jiang et al. [4] and He et al. [5], we may need to regard the small components as outliers rather than a cluster. Overall, in cases (b) and (c), it may be more difficult to say that the cluster size is exactly two than in case (a). This observation gives rise to the problem of formally defining the complexity of clustering structures that reflects the overlaps and weight biases.

This paper introduces a novel concept of *mixture complexity* (MC) to resolve this problem. It is related to the logarithm of the cluster size. For example, the exponentials of the MC are 2.00, 1.39, and 1.21 for cases (a), (b), and (c), respectively. In other words, given the mixture size, MC estimates the cluster size continuously rather than discretely.

There are two reasons for the need of MC. First, it theoretically evaluates the cluster size in the finite mixture model considering the overlap and imbalance between the components. Although their impacts on the cluster size have been discussed independently, we present a unified framework to interpret the cluster size with a continuous index. It presents a new perspective on model-based clustering and can be practically applied to cluster merging or clustering-based outlier detection. The second is the application of MC to the issue of gradual clustering change detection. Conventionally, clustering changes have been considered to be abrupt, induced by changes in the mixture size or cluster size. In reality, however, there are cases where mechanisms for generating data change gradually (or incrementally in the context of concept drifts [6]). We thereby present a new methodology for tracking such changes by observing MC’s changes.

We further show that MC can be used to quantify the cluster size in hierarchical mixture models. We demonstrate that the MC of a hierarchical mixture model can be decomposed into the sum of MCs for local mixture models. It enables us to evaluate the complexity of the substructures as well as the entire structure.

The concept of MC has been applied to the clustering merging problem in [7]. This study further investigates the theoretical properties of MC and proposes a new application for the issue of gradual clustering change detection.

### 1.2. Significance and Novelty

The significance and novelty of this paper are summarized below.

#### 1.2.1. Mixture Complexity for Finite Mixture Models

We introduce a novel concept of MC to continuously measure the cluster size in a mixture model. It is formally defined from the viewpoint of information theory and can be interpreted as a natural extension of the cluster size considering the overlaps and weight biases among the components. We further demonstrate that MC can be decomposed into a sum of MCs according to the mixture hierarchies; it helps us in analyzing MC in a decomposed manner.

#### 1.2.2. Applications of MC to Gradual Clustering Change Detection

We apply MC to the issue of monitoring gradual changes in clustering structures. We propose methods to monitor changes in MC instead of the mixture size or cluster size. Because MC takes a real value, it is more suitable for observing gradual changes. We empirically demonstrate that MC elucidates the clustering structures and their changes more effectively than the mixture size or cluster size.

The remainder of this paper is organized as follows. Section 2 discusses related work. In Section 3, we introduce the concept of MC and present some examples. Theoretical properties of MC are shown in Section 4. Section 5 discusses the application of MC to clustering change detection problems and Section 6 describes the experimental results. Finally, Section 7 concludes this paper. Proofs of the propositions and theorems are described in Appendices. Programs for the experiments are available at https://github.com/ShunkiKyoya/MixtureComplexity, accessed on 17 August 2022.

## 2. Related Work

The issue of determining the best mixture size or cluster size (often referred to as model selection) has extensively been studied. For example, AIC [8], BIC [9], and MDL [10] have been used to select the mixture size; ICL [11] and MDL-based clustering criteria [12,13] have been invented to select the cluster size. These methods have conventionally considered the cluster size as the same as the mixture size by regarding one mixture component as one independent cluster. See also a recent review by McLachlan and Rathnayake [14] focusing on the number of components in a Gaussian mixture model.

Differences between the mixture size and cluster size have also been widely discussed. For example, McLachlan and Peel [1] pointed out that there were cases that Gaussian mixture models with more than one mixture sizes were needed to describe one skewed cluster; Biernacki et al. [11] argued that in many situations, the mixture size estimated by BIC was too large to regard it as the cluster size. The problem of estimating the cluster size under a given mixture size has also been investigated by Hennig [3]; he proposed methods to identify the cluster structure by merging heavily overlapped mixture components. MC differs from his approach in that it interprets the clustering structure by only measuring the overlap rate rather than deciding whether to merge based on a certain threshold.

The degree of overlap or closeness between components was evaluated using various measures, such as the classification error rate or the Bhattacharyya distance [15]. Wang and Sun [16] and Sun and Wang [17] formulated the overlap rate of Gaussian distributions from the geometric nature of them. All of the works above have been limited to the case of two components. On the other hand, MC considers the overlap between any number of components.

Deciding whether a small component is a cluster or a set of outliers is also a significant matter. For example, clustering algorithms such as DBSCAN [18] and constrained *k*-means [19] avoided generating small components to obtain a better clustering structure. Jiang et al. [4] and He et al. [5] associated the small components with outlier detection problems. MC evaluates the small components by continuously measuring the impacts on the cluster size.

Some other notions have been proposed to quantify the clustering structure. Fuzzy clustering [20] is also a method used to estimate the clustering structures with cluster overlap; however, MC is more suitable for consistent estimation in that it assumes the background mixture distributions. Rusch et al. [21] evaluated the crowdedness of the data under the concept of “clusteredness”. However, its relations to the cluster size are indirect. Recently, descriptive dimensionality (Ddim) [22] was proposed to define the model dimensionality continuously. It can be implemented to estimate the clustering structure under the assumption of model fusion, that is, models with a different number of components are probabilistically mixed. MC differs from Ddim because it evaluates the overlap and weight bias in the single model without model fusion.

Clustering under the data stream has been discussed with various objectives [23,24,25]. We consider the problem of detecting changes in the cluster structure; Dynamic model selection (DMS) [26,27,28] addressed this problem by observing the changes in the models (corresponding to mixture size or cluster size in this paper). Because the models are valued discretely, the detected changes have been considered to be abrupt. Refer also to the notions of tracking best experts [29], evolution graph [30], and switching distributions [31], which are similar to DMS.

Furthermore, the issues of gradual changes have been discussed to investigate the transition periods for absolute changes. The MDL change statistics [32] and differential MDL change statistics [33] were proposed to measure the degree of gradual changes. The notions of structural entropy [34] and graph entropy [35] were proposed to measure the degree of model uncertainty in the changes. This study quantifies the degree of gradual changes using the fluctuations in MC and presents a new methodology to detect them.

MC is based on the mutual information between the observed and latent variables, which has been considered in the clustering fields. For example, Still et al. [36] regarded clustering as data compression and applied mutual information to measure its degree. In this paper, we present a novel interpretation of mutual information as a continuous number of clusters. Furthermore, we also present its novel applications for interpreting clusterings and clustering change detection.

## 3. Mixture Complexity

In this section, we formally introduce the mixture complexity and describe its properties using some examples and theories.

### 3.1. Definitions

Given the data ${\left\{x_{n}\right\}}_{n=1}^{N}$ and the finite mixture model *f* that have generated them, we consider interpreting the cluster size of *f*. The distribution *f* is written as

$$f\left(x\right):=\sum_{k=1}^{K}\rho_{k}g_{k}\left(x\right),$$

where *K* denotes the mixture size, ${\left\{\rho_{k}\right\}}_{k=1}^{K}$ denote the proportions of each component summing up to one, and ${\left\{g_{k}\right\}}_{k=1}^{K}$ denotes the probability distributions. The random variable *X* following the distribution *f* is called an *observed variable* because it can be observed as a datum. We also define the *latent variable* Z∈{1,…,K} as the index of the component from which the observed variable *X* originated. The pair (X,Z) is called a *complete variable*. The distribution of the latent variable P(Z) and the conditional distribution of the observed variable P(X|Z) can be given by

$$\begin{array}{cc} P(Z=k) & =\rho_{k},\\ P(X|Z=k) & =g_{k}\left(X\right). \end{array}$$

To investigate the clustering structures in *f*, we consider the following quantity:

I(Z;X):=H(Z)−H(Z|X),

where H(Z) and H(Z|X) denote the entropy and conditional entropy, respectively, of the latent variable *Z* defined as

$$\begin{array}{c} H\left(Z\right):=-\sum_{k=1}^{K}P\left(Z=k\right)\log P\left(Z=k\right)=-\sum_{k=1}^{K}\rho_{k}\log\rho_{k},\\ H\left(Z|X\right):=-E_{X}\left[\sum_{k=1}^{K}P\left(Z=k|X\right)\log P\left(Z=k|X\right)\right]=-E_{X}\left[\sum_{k=1}^{K}\gamma_{k}\left(X\right)\log\gamma_{k}\left(X\right)\right]. \end{array}$$

where

$$\gamma_{k}\left(X\right):=P\left(Z=k|X\right).$$

The quantity I(Z;X) is well-known as the mutual information between the observed and latent variables; it is also known as the (generalized) Jensen–Shannon Divergence [37]. We can interpret I(Z;X) as the volume of cluster structures as follows. Because I(Z;X) is a subtraction of the latent variable’s entropy with and without the knowledge of the observed variable, it represents the amount of information about the latent variable possessed by the observed data. Thus, its exponent exp(I(X;Z)) denotes the number of the latent variables distinguished by the observed variable; it can be interpreted as a continuous extension of the cluster size. For more information about entropy and mutual information, see the book written by Cover and Thomas [38].

However, I(Z;X) cannot be calculated analytically even if *f* is known. Thus, noting that $\rho_{k}=E_{X}\left[\gamma_{k}\left(X\right)\right]$, we approximate I(Z;X) using the data ${\left\{x_{n}\right\}}_{n=1}^{N}$ as follows:

$$I\left(Z;X\right)\approx \tilde{H}\left(Z\right)-\tilde{H}\left(Z|X\right),$$

where

$$\begin{array}{c} \tilde{H}\left(Z\right):=-\sum_{k=1}^{K}{\tilde{\rho }}_{k}\log{\tilde{\rho }}_{k},\\ \tilde{H}\left(Z|X\right):=-\frac{1}{N}\sum_{n=1}^{N}\sum_{k=1}^{K}\gamma_{k}\left(x_{n}\right)\log\gamma_{k}\left(x_{n}\right),\\ {\tilde{\rho }}_{k}:=\frac{1}{N}\sum_{n=1}^{N}\gamma_{k}\left(x_{n}\right). \end{array}$$

We call this the MC of the mixture model *f*.

**Definition** **1.** *Given the posterior probabilities ${\left\{\gamma_{k}\left(x_{n}\right)\right\}}_{k,n}$, we define the mixture complexity (MC) as*

$$\begin{array}{c} \mathrm{MC}\left({\left\{\gamma_{k}\left(x_{n}\right)\right\}}_{k,n}\right):=-\sum_{k=1}^{K}{\tilde{\rho }}_{k}\log{\tilde{\rho }}_{k}+\frac{1}{N}\sum_{n=1}^{N}\sum_{k=1}^{K}\gamma_{k}\left(x_{n}\right)\log\gamma_{k}\left(x_{n}\right), \end{array}$$

*where*

$$\begin{array}{c} {\tilde{\rho }}_{k}:=\frac{1}{N}\sum_{n=1}^{N}\gamma_{k}\left(x_{n}\right). \end{array}$$

*If the data have weights ${\left\{w_{n}\right\}}_{n}$, we define the MC as*

$$\begin{array}{c} \mathrm{MC}\left({\left\{\gamma_{k}\left(x_{n}\right)\right\}}_{k,n};{\left\{w_{n}\right\}}_{n}\right):=-\sum_{k=1}^{K}{\tilde{\rho }}_{k}\log{\tilde{\rho }}_{k}+\frac{1}{\sum_{n^{\prime }}w_{n^{\prime }}}\sum_{n=1}^{N}w_{n}\sum_{k=1}^{K}\gamma_{k}\left(x_{n}\right)\log\gamma_{k}\left(x_{n}\right), \end{array}$$

*where*

$$\begin{array}{c} {\tilde{\rho }}_{k}:=\frac{1}{\sum_{n^{\prime }}w_{n^{\prime }}}\sum_{n=1}^{N}w_{n}\gamma_{k}\left(x_{n}\right). \end{array}$$

The weighted version of MC is defined for later use.

Note that there are other ways to approximate I(Z;X); we adopt the form of Definition 1 because it has the decomposition property shown in Section 4.2. See also the methods used to approximate the entropy of the mixture model [39,40] that can also be applied to approximate I(Z;X).

In practice, only the data ${\left\{x_{n}\right\}}_{n=1}^{N}$ can be obtained without the underlying distribution *f*. Then, we estimate the posterior probabilities ${\left\{{\hat{\gamma }}_{k}\left(x_{n}\right)\right\}}_{k,n}$ from the data ${\left\{x_{n}\right\}}_{n=1}^{N}$ and further estimate the MC as

$$\mathrm{MC}\left({\left\{\gamma_{k}\left(x_{n}\right)\right\}}_{k,n}\right)\approx \mathrm{MC}\left({\left\{{\hat{\gamma }}_{k}\left(x_{n}\right)\right\}}_{k,n}\right).$$

It can be calculated even if the model *f* cannot be estimated.

### 3.2. Examples

In this subsection, we discuss some examples of MC to understand its notions.

#### 3.2.1. MC with Different Overlaps

First, we set N=600 and generated the data $x_{1},\ldots ,x_{600}\in R^{2}$ as follows.

$$x_{n}∼\left\{\begin{array}{cc} N\left(x_{n}{|\mu =\left[0,0\right]}^{⊤},\Sigma =I_{2}\right) & (1\leq n\leq 300),\\ N\left(x_{n}{|\mu =\left[\alpha ,0\right]}^{⊤},\Sigma =I_{2}\right) & (301\leq n\leq 600), \end{array}\right.$$

where N(x|μ,Σ) denotes a multivariate normal distribution with mean μ and covariance Σ, $I_{d}$ denotes a *d*-dimensional identity matrix, and α∈R is the parameter that determines the degree of overlap between two components.

**Figure 2:**
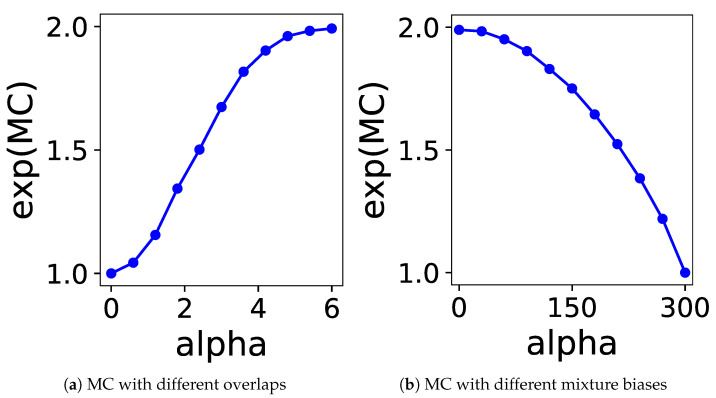
Relation between the parameter α and the exponential of the MC.

By varying the value of α among 0, 0.6, …, 6.0, we generated the data and measured the MC by setting $\rho_{1},\rho_{2}=1/2$ and $g_{1},g_{2}$ as the actual distributions. The exponential of the MC for each α is plotted in Figure 2a. It is evident from the figure that the MC smoothly increases from 1.0 to 2.0 as the two components become isolated.

#### 3.2.2. MC with Different Mixture Biases

Next, we set N=600 and generated the data $x_{1},\ldots ,x_{600}\in R^{2}$ as follows:

$$x_{n}∼\left\{\begin{array}{cc} N\left(x_{n}{|\mu =\left[0,0\right]}^{⊤},\Sigma =I_{2}\right) & (1\leq n\leq 300+\alpha ),\\ N\left(x_{n}{|\mu =\left[6,0\right]}^{⊤},\Sigma =I_{2}\right) & (301+\alpha \leq n\leq 600), \end{array}\right.$$

where α∈{0,…,300} is the parameter that determines the degree of bias between the proportion of two components.

By varying α among 0, 30, …, 300, we generated the data and measured the MC by setting $\rho_{1}=\left(300+\alpha \right)/600,\rho_{2}=\left(300-\alpha \right)/600$ and $g_{1},g_{2}$ as the actual distributions. The exponential of the MC for each α is plotted in Figure 2b. It is evident from the figure that the MC smoothly decreases from 2.0 to 1.0 as the balance becomes biased.

## 4. Theoretical Properties

In this subsection, we discuss the theoretical properties of MC.

### 4.1. Basic Properties

We discuss the basic properties of MC. The proofs are described in Appendix A.

First, we discuss the minimum and maximum of MC. We show that MC takes the minimum when the components entirely overlap and maximum when they are entirely separate.

**Proposition** **1.** *If the components entirely overlap, i.e., there exists $\gamma_{1},\ldots ,\gamma_{K}$ such that $\gamma_{k}\left(x_{n}\right)=\gamma_{k}$ for all k and n, then,*

$$\mathrm{MC}\left({\left\{\gamma_{k}\left(x_{n}\right)\right\}}_{k,n};{\left\{w_{n}\right\}}_{n}\right)=0.$$

**Proposition** **2.** *If the components are entirely separate, i.e., for all $x_{n}$, there is a unique index $k_{n}$ that satisfies*

$$\gamma_{l}\left(x_{n}\right)=\left\{\begin{array}{cc} 1 & \left(l=k_{n}\right)\\ 0 & \left(l\neq k_{n}\right) \end{array}\right.,$$

*then,*

$$\mathrm{MC}\left({\left\{\gamma_{k}\left(x_{n}\right)\right\}}_{k,n};{\left\{w_{n}\right\}}_{n}\right)=\tilde{H}\left(Z\right).$$

*In particular, if the components are entirely balanced, i.e., ${\tilde{\rho }}_{1}=\ldots ={\tilde{\rho }}_{K}=1/K$, then,*

$$\mathrm{MC}\left({\left\{\gamma_{k}\left(x_{n}\right)\right\}}_{k,n};{\left\{w_{n}\right\}}_{n}\right)=\log K.$$

**Proposition** **3.** *For all ${\left\{\gamma_{k}\left(x_{n}\right)\right\}}_{k,n}$, MC satisfies*

$$0\leq \mathrm{MC}\left({\left\{\gamma_{k}\left(x_{n}\right)\right\}}_{k,n};{\left\{w_{n}\right\}}_{n}\right)\leq \log K.$$

*Moreover, MC takes 0 only if the components are entirely overlapping as stated in Proposition 1 and takes logK only if the components are entirely separate as stated in Proposition 2.*

Next, we show that the value of MC is invariant with the representation of the mixture distribution. For example, consider the following three mixture distributions:

$$\begin{array}{cc} f_{1}\left(x\right)\; & =\frac{1}{2}g_{1}\left(x\right)+\frac{1}{2}g_{2}\left(x\right),\\ f_{2}\left(x\right)\; & =\frac{1}{2}g_{1}\left(x\right)+\frac{1}{4}g_{2}\left(x\right)+\frac{1}{4}g_{2}\left(x\right),\\ f_{3}\left(x\right)\; & =\frac{1}{2}g_{1}\left(x\right)+\frac{1}{4}g_{2}\left(x\right)+\frac{1}{4}g_{2}\left(x\right)+0\cdot g_{3}\left(x\right). \end{array}$$

In $f_{2}$ and $f_{3}$, we need to manually remove the redundant components and regard the mixture size as two [1]. On the other hand, the following property indicates that the MCs for $f_{1},f_{2}$, and $f_{3}$ are the same; thus, we need not to care about their differences in evaluating MC.

**Proposition** **4.** *If there exists a set $I_{1},\ldots ,I_{L},I_{\infty }$ that partitions {1,…,K} and distributions $g_{1}^{0},\ldots ,g_{L}^{0}$ such that*

$$\begin{array}{c} k\in I_{l}\Rightarrow g_{k}=g_{l}^{0}\;\left(l=1,\ldots ,L\right),\\ k\in I_{\infty }\Rightarrow \rho_{k}=0, \end{array}$$

*then*

$$\mathrm{MC}\left({\left\{\gamma_{k}\left(x_{n}\right)\right\}}_{k,n};{\left\{w_{n}\right\}}_{n}\right)=\mathrm{MC}\left({\left\{\gamma_{l}^{0}\left(x_{n}\right)\right\}}_{l,n};{\left\{w_{n}\right\}}_{n}\right),$$

*where*

$$\gamma_{l}^{0}\left(x\right)=\frac{\left(\sum_{k\in I_{l}}\rho_{k}\right)g_{l}^{0}\left(x\right)}{f^{0}\left(x\right)},\;f^{0}\left(x\right)=\sum_{l=1}^{L}\left(\sum_{k\in I_{l}}\rho_{k}\right)g_{l}^{0}\left(x\right).$$

### 4.2. Decomposition Property

In this section, we discuss a method to decompose MC along the hierarchies in mixture models; this can help us in analyzing the structures in more detail.

**Figure 3:**
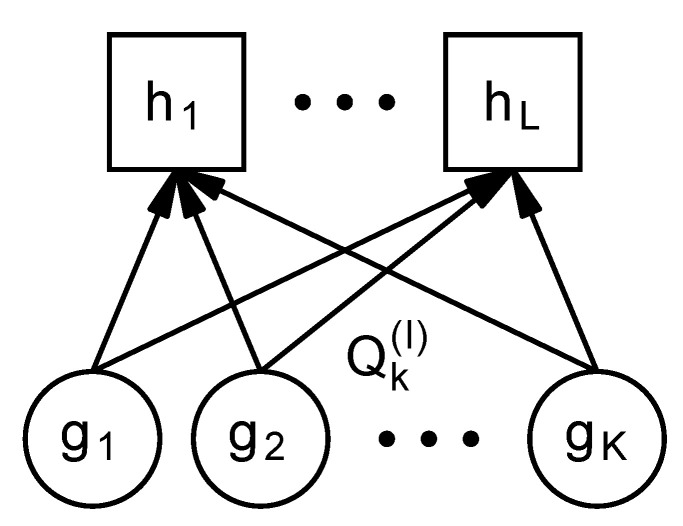
Hierarchy in the mixture model.

Consider that the mixture distribution *f* has a two-stage hierarchy, as shown in Figure 3. It has *K* components ${\left\{g_{k}\right\}}_{k=1}^{K}$ on the lower side and *L* components ${\left\{h_{l}\right\}}_{l=1}^{L}$ on the upper side, where ${\left\{g_{k}\right\}}_{k=1}^{K}$ denote the probability distributions and ${\left\{h_{l}\right\}}_{l=1}^{L}$ denote their mixture distributions, respectively. We construct the hierarchy as follows. First, we estimate the distribution $f=\sum_{k=1}^{K}\rho_{k}g_{k}$. Then, we obtain ${\left\{h_{l}\right\}}_{l=1}^{L}$ by partitioning (or clustering) the lower components into *L* groups. Formally, we denote $Q_{k}^{\left(l\right)}\in R_{\geq 0}$ as the proportion of the lower component k∈{1,…,K} that belongs to the upper component *l*, which satisfies $\sum_{l=1}^{L}Q_{k}^{\left(l\right)}=1$ for all *k*. Then, we derive ${\left\{h_{l}\right\}}_{l=1}^{L}$ by rewriting $f=\sum_{k=1}^{K}\rho_{k}g_{k}$ as

$$f\left(x\right)=\sum_{k=1}^{K}\rho_{k}g_{k}\left(x\right)=\sum_{k=1}^{K}\sum_{l=1}^{L}Q_{k}^{\left(l\right)}\rho_{k}g_{k}\left(x\right)=\sum_{l=1}^{L}\tau_{l}h_{l}\left(x\right),$$

where

$$\begin{array}{c} \tau_{l}:=\sum_{k=1}^{K}Q_{k}^{\left(l\right)}\rho_{k},\;h_{l}\left(x\right):=\sum_{k=1}^{K}\rho_{k}^{\left(l\right)}g_{k}\left(x\right),\;\rho_{k}^{\left(l\right)}:=\frac{Q_{k}^{\left(l\right)}\rho_{k}}{\sum_{k^{\prime }}Q_{k^{\prime }}^{\left(l\right)}\rho_{k^{\prime }}}. \end{array}$$

According to the hierarchy, we can decompose the MC.

**Theorem** **1.** *We can decompose the MC as follows:*

$$\begin{array}{cc} \; & \;\mathrm{MC}\left({\left\{\gamma_{k}\left(x_{n}\right)\right\}}_{k,n};{\left\{w_{n}\right\}}_{n}\right)\\ = & \;\mathrm{MC}\left({\left\{\sum_{k=1}^{K}Q_{k}^{\left(l\right)}\gamma_{k}\left(x_{n}\right)\right\}}_{l,n};{\left\{w_{n}\right\}}_{n}\right)+\sum_{l=1}^{L}W_{l}\cdot \mathrm{MC}\left({\left\{\gamma_{k}^{\left(l\right)}\left(x_{n}\right)\right\}}_{k,n};{\left\{w_{n}^{\left(l\right)}\right\}}_{n}\right), \end{array}$$

*where*

$$\begin{array}{c} W_{l}=\frac{\sum_{n}w_{n}\sum_{k}Q_{k}^{\left(l\right)}\gamma_{k}\left(x_{n}\right)}{\sum_{n^{\prime }}w_{n^{\prime }}}=\sum_{k=1}^{K}Q_{k}^{\left(l\right)}{\tilde{\rho }}_{k},\\ w_{n}^{\left(l\right)}=w_{n}\sum_{k=1}^{K}Q_{k}^{\left(l\right)}\gamma_{k}\left(x_{n}\right),\\ \gamma_{k}^{\left(l\right)}\left(x_{n}\right)=\frac{Q_{k}^{\left(l\right)}\gamma_{k}\left(x_{n}\right)}{\sum_{k^{\prime }}Q_{k^{\prime }}^{\left(l\right)}\gamma_{k^{\prime }}\left(x_{n}\right)}. \end{array}$$

The proof is described in the Appendix B. For notational simplicity, we will use the following terms:

$$\begin{array}{cc} \mathrm{MC}(\mathrm{total}):= & \mathrm{MC}\left({\left\{\gamma_{k}\left(x_{n}\right)\right\}}_{k,n};\left\{w_{n}\right\}\right),\\ \mathrm{MC}(\mathrm{interaction}):= & \mathrm{MC}\left({\left\{\sum_{k}Q_{k}^{\left(l\right)}\gamma_{k}\left(x_{n}\right)\right\}}_{l,n};{\left\{w_{n}\right\}}_{n}\right),\\ \mathrm{Contribution}(\mathrm{component}\;l):= & W_{l}\cdot \mathrm{MC}\left({\left\{\gamma_{k}^{\left(l\right)}\left(x_{n}\right)\right\}}_{k,n};{\left\{w_{n}^{\left(l\right)}\right\}}_{n}\right),\\ W(\mathrm{component}\;l):= & W_{l},\\ \mathrm{MC}(\mathrm{component}\;l):= & \mathrm{MC}\left({\left\{\gamma_{k}^{\left(l\right)}\left(x_{n}\right)\right\}}_{k,n};{\left\{w_{n}^{\left(l\right)}\right\}}_{n}\right). \end{array}$$

Then, we can rewrite Theorem 1 as

$$\begin{array}{c} \mathrm{MC}(\mathrm{total})=\mathrm{MC}(\mathrm{interaction})+\sum_{l=1}^{L}\mathrm{Contribution}(\mathrm{component}\;l),\\ \mathrm{Contribution}(\mathrm{component}\;l)=W(\mathrm{component}\;l)\cdot \mathrm{MC}(\mathrm{component}\;l). \end{array}$$

In Theorem 1, the MC of the entire structure (MC(total)) is decomposed into a sum of the MC among the upper components (MC(interaction)) and their respective contributions (Contribution(component *l*)). Contribution(component *l*) is further decomposed into a product of the weight (W(component *l*)) and complexity (MC(component *l*)) of the component. Because $w_{n}^{\left(l\right)}$ denotes the weight of $x_{n}$ that belongs to component *l*, its sum W(component *l*) represents the total weights of the data contained in it. Additionally, MC(component *l*) denotes the clustering structures in component *l* considering the data weights.

**Figure 4:**
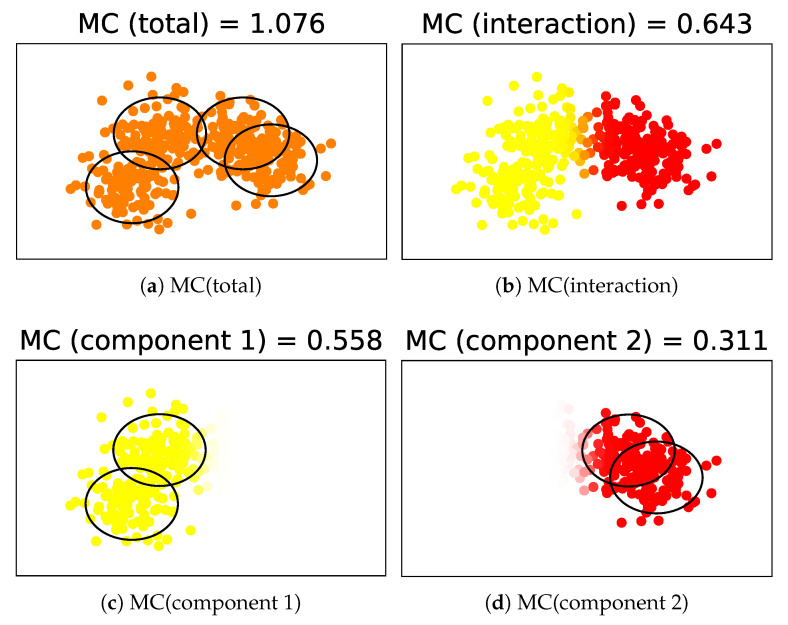
Example of the decomposition of MC. The data’s color in (**b**) and thickness in (**c**,**d**) correspond to the data weights $w_{n}^{\left(l\right)}$.

An example of the decomposition is illustrated in Figure 4 and Table 1. In this example, there are K=4 lower components generated from a Gaussian mixture model; additionally, there are L=2 upper components on the left and right sides. By decomposing MC(total), we can evaluate the complexities in the local structures as well as those in the entire structure.

### 4.3. Consistency

In this subsection, we discuss the consistency of the MC: as the estimated distribution becomes close to the true distribution, the estimated MC also converges to the true value. Formally, we define the set of *K*-component mixture models as

$$F_{K}:=\left\{f\left(x\right)=\sum_{k=1}^{K}\rho_{k}g\left(x|\theta_{k}\right)\;|\;\rho_{1},\ldots ,\rho_{K}\geq 0,\;\sum_{k=1}^{K}\rho_{k}=1,\;\theta_{1},\ldots ,\theta_{K}\in \Theta \right\}.\;$$

We assume that the space $F_{K}$ is weakly identifiable, that is,

$$\sum_{k=1}^{K}\rho_{k}^{0}g\left(\cdot |\theta_{k}^{0}\right)=\sum_{k=1}^{K}\rho_{k}^{1}g\left(\cdot |\theta_{k}^{1}\right)\;\Leftrightarrow \;\sum_{k=1}^{K}\rho_{k}^{0}\delta_{\Theta }\left(\cdot =\theta_{k}^{0}\right)=\sum_{k=1}^{K}\rho_{k}^{1}\delta_{\Theta }\left(\cdot =\theta_{k}^{1}\right),\;$$

where $\delta_{\Theta }$ is the Kronecker’s delta function on Θ. This condition states that the same distributions should have the same mixtures of parameters. See Teicher [41] and Yakiwitz and Spragins [42] for sufficient conditions on this kind of identifiability; in their work, it has been shown that this is satisfied in Gaussian or gamma mixtures.

We also assume some true mixture distribution written as

$$f^{☆}\left(x\right)=\sum_{k=1}^{K^{☆}}\rho_{k}^{☆}g\left(x|\theta_{k}^{☆}\right),\;\rho_{1}^{☆},\ldots ,\rho_{K^{☆}}^{☆}>0,\;\theta_{1}^{☆},\ldots ,\theta_{K^{☆}}^{☆}\in \Theta ,\;\theta_{i}^{☆}\neq \theta_{j}^{☆}\;\left(i\neq j\right)\;$$

generates the data $x^{N}$. We consider estimating the true mixture complexity written as $\mathrm{MC}({\left\{\gamma_{k}^{☆}\left(x_{n}\right)\right\}}_{k,n})$ by substituting the estimated distribution $f\in F_{K}$ into $f^{☆}$. We restrict our analysis to the case that $K\geq K^{☆}$ so that $F_{K}$ contains distributions that are equivalent to $f^{☆}$. Then, we show that $\mathrm{MC}({\left\{\gamma_{k}\left(x_{n}\right)\right\}}_{k,n})$ converges to $\mathrm{MC}({\left\{\gamma_{k}^{☆}\left(x_{n}\right)\right\}}_{k,n})$ as *f* and $f^{☆}$ become closer.

To analyze the convergence, we re-parametrize the estimated parameters using the method proposed in Liu and Shao [43]. They note that if $f=f^{☆}$, there exist integers $0=i_{0}<\ldots <i_{K^{☆}}\leq K$ such that the following holds under some permutation of the components:

$$\left\{\begin{array}{cc} \theta_{l}=\theta_{k}^{☆} & \left(l\in I_{k},k=1,\ldots ,K^{☆}\right),\\ \rho_{k}=0 & \left(k\in I_{\infty }\right), \end{array}\right.$$

where

$$\begin{array}{cc} I_{k}:= & \left\{i_{k-1}+1,\ldots ,i_{k}\right\}\;\left(k=1,\ldots ,K^{☆}\right),\\ I_{\infty }:= & \left\{i_{K^{☆}}+1,\ldots ,K\right\}. \end{array}$$

Then, they parametrize the parameters in *f* using two kinds of parameters defined as

$$\begin{array}{cc} \phi := & \left({\left\{\theta_{k}\right\}}_{k=1}^{i_{K^{☆}}},{\left\{r_{l}\right\}}_{l=1}^{K^{☆}},{\left\{\rho_{k}\right\}}_{k=i_{K^{☆}}+1}^{K}\right),\\ r_{l}:= & \sum_{k\in I_{l}}\rho_{k},\\ \psi := & \left({\left\{s_{k}\right\}}_{k=1}^{i_{K^{☆}}},{\left\{\theta_{k}\right\}}_{k=i_{K^{☆}}+1}^{K}\right),\\ s_{k}:= & \frac{\rho_{k}}{r_{k}}\;\left(k\in I_{l}\right) \end{array}$$

and rewrite *f* as

$$\begin{array}{cc} f(x)\; & =\sum_{k=1}^{K}\rho_{k}g\left(x|\theta_{k}\right)=\sum_{l=1}^{K^{☆}}r_{l}h_{l}\left(x\right)+\sum_{k=i_{K^{☆}}+1}^{K}\rho_{k}g_{k}\left(x\right),\\ h_{l}\left(x\right)\; & =\sum_{k\in I_{l}}s_{k}g_{k}\left(x\right). \end{array}$$

In this parametrization, $f=f^{☆}$ is equivalent to

$$\phi =\phi^{☆}:=\left(\left\{\theta_{1}^{☆},\ldots ,\theta_{1}^{☆},\ldots ,\theta_{K^{☆}}^{☆},\ldots ,\theta_{K^{☆}}^{☆}\right\},\left\{\rho_{1}^{☆},\ldots ,\rho_{K^{☆}}^{☆}\right\},\left\{0,\ldots ,0\right\}\right);$$

the parameter ψ has nothing to do with equivalence. This parametrization represents two types of convergence in mixture models. First, it overlaps the components to the true distributions, which is realized by

$$\begin{array}{cc} {\left\{\theta_{k}\right\}}_{k=1}^{i_{K^{☆}}}\; & \to \left\{\theta_{1}^{☆},\ldots ,\theta_{1}^{☆},\ldots ,\theta_{K^{☆}}^{☆},\ldots ,\theta_{K^{☆}}^{☆}\right\},\\ {\left\{r_{l}\right\}}_{l=1}^{K^{☆}}\; & \to \left\{\rho_{1}^{☆},\ldots ,\rho_{K^{☆}}^{☆}\right\}. \end{array}$$

The other is shrinking the weights of the redundant components to zero, which is realized by

$${\left\{\rho_{k}\right\}}_{k=i_{K^{☆}}+1}^{K}\to \left\{0,\ldots ,0\right\}.$$

We use the following conditions for our proof:

(C1) g(·|θ) is differentiable once and for every $k=1,\ldots ,K^{☆}$ and there exists ϵ>0 such that
  $$E_{\theta_{k}^{☆}}\left[\underset{\theta :∥\theta -\theta_{k}^{☆}∥\leq \epsilon }{\sup}∥\frac{\nabla g(\cdot |\theta )}{g(\cdot |\theta )}∥\right]<\infty .$$
(C2) As N→∞, the estimated parameter ϕ satisfies
  $$∥\phi -\phi^{☆}∥=o_{P}\left(1\right).$$
(C3) Let us define the approximations of mixture proportions as
  $$\begin{array}{cc} {\tilde{r}}_{l}:= & \frac{1}{N}\sum_{n=1}^{N}\sum_{k\in I_{l}}\gamma_{k}\left(x_{n}\right)\;\left(l=1,\ldots ,K^{☆}\right),\\ {\tilde{\rho }}_{\infty }:= & \frac{1}{N}\sum_{n=1}^{N}\sum_{k=i_{K^{☆}}+1}^{K}\gamma_{k}\left(x_{n}\right). \end{array}$$
  Then, as N→∞, they satisfy
  $$\begin{array}{cc} \left|{\tilde{r}}_{l}-\rho_{l}^{☆}\right|\; & =o_{P}\left(1\right)\;\left(l=1,\ldots ,K^{☆}\right),\\ {\tilde{\rho }}_{\infty }\; & =o_{P}\left(1\right). \end{array}$$

Condition (C1) is a usual differentiability condition, and (C2) and (C3) require consistency of the parameters. It is known that consistent estimations are possible by penalized maximum likelihood estimation [44,45] or Bayesian estimation [46], for example. Then, the consistency of the MC is shown as the following theorem.

**Theorem** **2.** *Under assumptions (C1), (C2), and (C3), the following holds as N→∞:*

$$\begin{array}{cc} \; & \left|\mathrm{MC}\left({\left\{\gamma_{k}\left(x_{n}\right)\right\}}_{k,n}\right)-\mathrm{MC}\left({\left\{\gamma_{k}^{☆}\left(x_{n}\right)\right\}}_{k,n}\right)\right|\\ = & \;O_{P}\left(∥\phi -\phi^{☆}∥+\sum_{l=1}^{K^{☆}}\left|{\tilde{r}}_{l}-\rho_{l}^{☆}\right|+{\tilde{\rho }}_{\infty }\log\left(-{\tilde{\rho }}_{\infty }\right)+\frac{1}{\sqrt{N}}\right). \end{array}$$

The proof is described in Appendix C. Theorem 2 shows the convergence rate of the estimation error of the MC. It is interesting that this even holds when $K\neq K^{☆}$. Therefore, it can be said that MC is a fundamental quantity to represent the cluster structures in mixture models by overcoming the differences in mixture size.

We discuss the overview of the proofs below. First, applying Theorem 1 repeatedly, we decompose the entire MC into the following four terms:

(a) Interaction between $\sum_{l=1}^{K^{☆}}r_{l}h_{l}$ and $\sum_{k=i_{K^{☆}}+1}^{K}\rho_{l}g_{l}$.
(b) Contribution from $\sum_{k=i_{K^{☆}}+1}^{K}\rho_{l}g_{l}$.
(c) Interaction among $h_{1},\ldots ,h_{K^{☆}}$
(d) Contributions from $h_{1},\ldots ,h_{K^{☆}}$, respectively.

**Figure 5:**
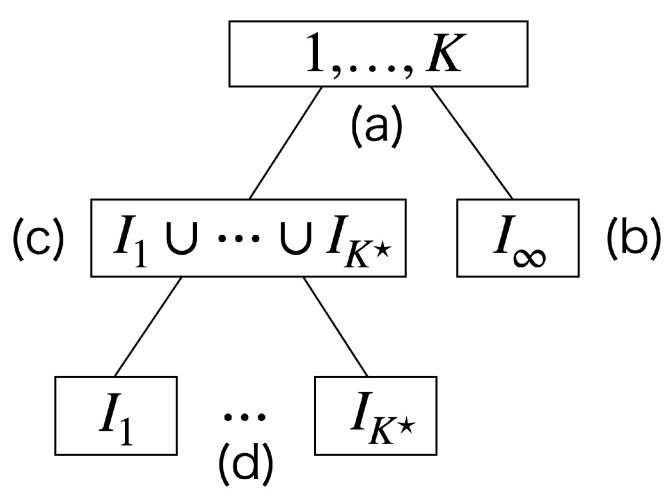
Decomposition of the MC to prove Theorem 2.

The procedure of the decomposition is also illustrated in Figure 5. Then, we show that

(a) tends to 0 because ${\tilde{\rho }}_{\infty }\to 0$;
(b) tends to 0 because ${\tilde{\rho }}_{\infty }\to 0$;
(c) tends to $\mathrm{MC}({\left\{\gamma_{k}^{☆}\left(x_{n}\right)\right\}}_{k,n})$ because $h_{1},\ldots ,h_{K^{☆}}$ tends to $g_{1},\ldots ,g_{K^{☆}}$;
(d) tends to 0 because for all *l*, all components in $h_{l}$ tends to $g_{l}$.

The proofs are mainly based on the mean-value theorem. However, differentiation of logf by $\rho_{k}\;\left(k\in I_{\infty }\right)$ may be infinite; we need additional treatments to avoid it.

## 5. Applications

In this section, we propose methods to apply the MC to clustering change detection problems. Formally speaking, given the dataset $X:=\{{\left\{x_{n,t}\right\}}_{n=1}^{N}|t\in 1,\ldots ,T\}$, where *t* denotes the time and ${\left\{x_{n,t}\right\}}_{n=1}^{N}$ denote the data generated at each *t*, we consider the problem of monitoring the changes in the clustering structures over t=1,…,T.

First, we briefly summarize the method named sequential dynamic model selection (SDMS) [28] that addresses this problem. Then, we introduce our ideas and discuss the differences between SDMS.

Hereafter, we assume that the data points $x_{n,t}$ are *d*-dimensional vectors and consider a Gaussian mixture model

$$f_{t}\left(x\right)=\sum_{k=1}^{K_{t}}\rho_{k,t}N\left(x|\mu_{k,t},\Sigma_{k,t}\right)$$

for each *t*.

### 5.1. Sequential Dynamic Model Selection

SDMS is an algorithm that is used to sequentially estimate models and find changes. In clustering change detection problems, it sequentially estimates the mixture sizes ${\hat{K}}_{t}$ and parameters $\eta_{{\hat{K}}_{t}}:={\left\{{\hat{\rho }}_{k,t},{\hat{\mu }}_{k,t},{\hat{\Sigma }}_{k,t}\right\}}_{k=1}^{{\hat{K}}_{t}}$ and finds model changes as changes in ${\hat{K}}_{t}$.

The estimation procedures are explained below. First, depending on the estimated mixture size at the last time point ${\hat{K}}_{t-1}$, we set the candidate for $K_{t}$. Then, for each $K_{t}$ in the candidate, we estimate the parameters $\theta_{K_{t}}$ from the data ${\left\{x_{n,t}\right\}}_{n=1}^{N}$ and calculate a cost function $L_{\mathrm{SDMS}}\left({\left\{x_{n,t}\right\}}_{n=1}^{N};K_{t},\theta_{K_{t}},{\hat{K}}_{t-1}\right)$. Finally, we select $K_{t}$ as the mixture size that minimizes the costs. The candidates of $K_{t}$ are set as

$$\left\{1,\ldots ,K_{\max}\right\}$$

at t=1, and

$$\left\{K_{t-1}-1,K_{t-1},K_{t-1}+1\right\}\cap \left\{1,\ldots ,K_{\max}\right\}$$

at t≥2, where $K_{\max}$ is a pre-defined parameter. The cost function denotes the sum of the code length functions of the model and model changes given by

$$L_{\mathrm{SDMS}}\left({\left\{x_{n,t}\right\}}_{n=1}^{N};K_{t},\eta_{K_{t}},{\hat{K}}_{t-1}\right)=L_{\mathrm{model}}\left({\left\{x_{n,t}\right\}}_{n=1}^{N};K_{t},\eta_{K_{t}}\right)+L_{\mathrm{change}}\left(K_{t}\;|\;{\hat{K}}_{t-1}\right).$$

#### Code Length of the Model

The score $L_{\mathrm{model}}\left({\left\{x_{n}\right\}}_{n=1}^{N};K,\eta_{K}\right)$ denotes a sum of the logarithm of the likelihood functions and penalty terms corresponding to the complexity of the model. In this study, we consider two likelihood functions and four penalty terms. For the (logarithm of) likelihood functions, we consider the *observed likelihood* $L({\left\{x_{n}\right\}}_{n=1}^{N};\theta_{K})$ and *complete likelihood* $L({\left\{x_{n},z_{n}\right\}}_{n=1}^{N};\theta_{K})$, provided by

$$\begin{array}{c} L\left({\left\{x_{n}\right\}}_{n=1}^{N};\theta_{K}\right):=\sum_{n=1}^{N}\log P\left(X=x_{n}\right)=\sum_{n=1}^{N}\log\left(\sum_{k=1}^{K}\rho_{k}N\left(x_{n}|\mu_{k},\Sigma_{k}\right)\right),\\ L\left({\left\{x_{n},z_{n}\right\}}_{n=1}^{N};\theta_{K}\right):=\sum_{n=1}^{N}\log P\left(X=x_{n},Z=z_{n}\right)=\sum_{n=1}^{N}\log\left(\rho_{z_{n}}N\left(x_{n}|\mu_{z_{n}},\Sigma_{z_{n}}\right)\right), \end{array}$$

where ${\left\{z_{n}\right\}}_{n=1}^{N}$ are the latent variables for the data estimated by

$$z_{n}:=\underset{z\in 1,\ldots ,K}{\mathrm{argmax}}P\left(Z=z|X=x_{n}\right).$$

They correspond to the likelihood of the observed data and complete data, respectively; the former is used to determine the mixture size, and the latter is used to determine the cluster size under the assumption that it is equal to the mixture size. For the penalty terms, we consider AIC [8], BIC [9], NML [13], and DNML [47,48]. By combining the log-likelihood and the penalty terms, we consider the following six scores:

- AIC with observed likelihood (AIC):
  $$-L\left({\left\{x_{n}\right\}}_{n=1}^{N};\eta_{K}\right)+D,$$
- AIC with complete likelihood (AIC+comp):
  $$-L\left({\left\{x_{n},z_{n}\right\}}_{n=1}^{N};\eta_{K}\right)+D,$$
- BIC with observed likelihood (BIC):
  $$-L\left({\left\{x_{n}\right\}}_{n=1}^{N};\eta_{K}\right)+\frac{D}{2}\log N,$$
- BIC with complete likelihood (BIC+comp):
  $$-L\left({\left\{x_{n},z_{n}\right\}}_{n=1}^{N};\eta_{K}\right)+\frac{D}{2}\log N,$$
- NML:
  $$-L\left({\left\{x_{n},z_{n}\right\}}_{n=1}^{N};\eta_{K}\right)+\log{\mathrm{PC}}_{\mathrm{NML}}\left(N,K\right),$$
- DNML:
  $$-L\left({\left\{x_{n},z_{n}\right\}}_{n=1}^{N};\theta_{K}\right)+\log{\mathrm{PC}}_{\mathrm{DNML}}\left(N,{\left\{z_{n}\right\}}_{n=1}^{N},K\right).$$

where D:=(K−1)+d(d+3)/2 denotes the number of the free parameters required to represent a Gaussian mixture model; ${\mathrm{PC}}_{\mathrm{NML}}\left(N,K\right)$ and ${\mathrm{PC}}_{\mathrm{DNML}}\left(N,{\left\{z_{n}\right\}}_{n=1}^{N},K\right)$ denote the parametric complexities. In our experiments, we estimated the parameter $\eta_{K}$ by conducting the EM algorithm [49] implemented in the Scikit-learn package [50] ten times and selected the best parameter that minimized each score. Note that in NML and DNML, we only considered the complete likelihood functions because only the methods to calculate their parametric complexities are known.

### 5.2. Track MC

In SDMS, clustering changes are detected as the changes of the mixture size or cluster size *K*; because it is discrete, the changes have been considered to be abrupt. Then, we propose to track MC instead of *K* while estimating the parameters using SDMS. Because MC takes a real value, monitoring it is more suitable for observing gradual changes than monitoring *K*. The algorithm for tracking MC is explained in Algorithm 1.
**Algorithm 1** Tracking MC**Require:** A dataset $X=\{{\left\{x_{n,t}\right\}}_{n=1}^{N}|t\in 1,\ldots ,T\}$.
1: **for** *t* = 1 **to** *t* = *T* **do**2:       Estimate ${\hat{K}}_{t}$ and ${\left\{{\hat{g}}_{k,t}\right\}}_{k=1}^{{\hat{K}}_{t}}$ from the data ${\left\{x_{n,t}\right\}}_{n=1}^{N}$ using SDMS.3:       Calculate ${\mathrm{MC}}_{t}:=\mathrm{MC}\left({\left\{{\hat{\gamma }}_{k}\left(x_{n}\right)\right\}}_{k,n}\right)$.4: **end for**5: **return** ${\left\{{\mathrm{MC}}_{t}\right\}}_{t=1}^{T}$.


### 5.3. Track MC with Its Decomposition

In addition to monitoring the MC of the entire structure, we also propose an algorithm to track its decomposition. To accomplish this, we must estimate the upper *L* components and their corresponding partitions $Q_{k,t}^{\left(l\right)}$ for each *t*.

Here, we assume that the upper *L* components are common at every *t* and estimate the partition $Q_{k,t}^{\left(l\right)}$ after estimating the lower components at each time. Specifically, we consider $\mu_{k,t}$ as a point with weights $\rho_{k,t}$ for each *k* and *t* and cluster them. As the clustering algorithm, we modified the fuzzy c-means [20] to handle the weighted points. Formally, we estimated the centers of the upper *L* components ${\tilde{\mu }}_{l}$ and their corresponding partitions $Q_{k,t}^{\left(l\right)}$ by minimizing the loss function

$$\sum_{t,k}\rho_{k,t}\sum_{l=1}^{L}{\left(Q_{k,t}^{\left(l\right)}\right)}^{m}{∥\mu_{k,t}-{\tilde{\mu }}_{l}∥}^{2},$$

where m>0 is parameter that determines the fuzziness of the partition.

We estimated ${\tilde{\mu }}_{l}$ and $Q_{k,t}^{\left(l\right)}$ by minimizing one iteratively while fixing another. We can formulate the iteration as follows:

$$\begin{array}{cc} {\tilde{\mu }}_{l}\; & =\frac{\sum_{k,t}\rho_{k,t}{\left(Q_{k,t}^{\left(l\right)}\right)}^{m}\mu_{k,t}}{\sum_{k^{\prime },t^{\prime }}\rho_{k^{\prime },t^{\prime }}{\left(Q_{k^{\prime },t^{\prime }}^{\left(l\right)}\right)}^{m}},\\ Q_{k,t}^{\left(l\right)}\; & =\frac{{∥\mu_{k,t}-{\tilde{\mu }}_{l}∥}^{2/(m-1)}}{\sum_{l^{\prime }=1}^{L}{∥\mu_{k,t}-{\tilde{\mu }}_{l^{\prime }}∥}^{2/(m-1)}}. \end{array}$$

Finally, we present an algorithm to track the MC and its decomposition in Algorithm 2. We can analyze the structural changes in more detail by evaluating the decomposed values.
**Algorithm 2** Tracking MC with its decomposition**Require:** A dataset $X=\{{\left\{x_{n,t}\right\}}_{n=1}^{N}|t\in 1,\ldots ,T\}$, parameters *m* and *L*.
1:2: *# Step 1: Estimate lower components.*3: **for** t=1 **to** t=T **do**4:       Estimate ${\hat{K}}_{t}$ and ${\left\{{\hat{g}}_{k,t}\right\}}_{k=1}^{{\hat{K}}_{t}}$ from the data ${\left\{x_{n,t}\right\}}_{n=1}^{N}$ using SDMS.5:       Calculate ${\mathrm{MC}(\mathrm{total})}_{t}:=\mathrm{MC}\left({\left\{{\hat{\gamma }}_{k}\left(x_{n}\right)\right\}}_{k,n}\right)$.6: **end for**7:8: *# Step 2: Estimate upper components and partition.*9: Estimate the centers ${\tilde{\mu }}_{l}$ and the partition $Q_{k,t}^{\left(l\right)}$ using fuzzy c-means.10:11: *# Step 3: Calculate the decomposition of MC.*12: **for** t=1 **to** t=T **do**13:       Calculate ${\mathrm{MC}(\mathrm{interaction})}_{t}$ defined in Section 4.2.14:       **for** l=1 **to** l=L **do**15:           Calculate $W(\mathrm{component}\;l{)}_{t}$ defined in Section 4.2.16:           Calculate $\mathrm{MC}(\mathrm{component}\;l{)}_{t}$ defined in Section 4.2.17:     **end for**18: **end for**19: **return** ${\left\{{\mathrm{MC}(\mathrm{total})}_{t}\right\}}_{t=1}^{T}$, ${\left\{{\mathrm{MC}(\mathrm{interaction})}_{t}\right\}}_{t=1}^{T}$, ${\left\{{\left\{W(\mathrm{component}\;l{)}_{t}\right\}}_{l=1}^{L}\right\}}_{t=1}^{T}$, ${\left\{{\left\{\mathrm{MC}(\mathrm{component}\;l{)}_{t}\right\}}_{l=1}^{L}\right\}}_{t=1}^{T}$.


## 6. Experimental Results

In this section, we present the experimental results that demonstrate the MC’s ability to monitor the clustering changes. We compare our methods to the monitoring of *K*.

### 6.1. Analysis of Artificial Data

To reveal the behaviors of MC, we conducted experiments with two artificial datasets called *move Gaussian dataset* and *imbalance Gaussian dataset*. Their experimental designs are discussed below. First, we generated artificial datasets $X=\{{\left\{x_{n,t}\right\}}_{n=1}^{N}|t\in 1,\ldots ,T\}$ by setting T=150 and N=1000. The datasets have one transaction period t=51,…,100 in which the data change their clustering structures gradually. Then, we estimated the MC and *K* using the methods in Section 5.1 and Section 5.2 by setting $K_{\max}=10$. To compare them, we first created a simple algorithm to detect the changes from the sequence of MC or *K*. Then, we compared the abilities of this algorithm in terms of the speed and accuracy of detecting the change points. Moreover, to evaluate the abilities to find the changes in the opposite direction, we performed experiments with the same datasets in the reverse order.

Given a sequence of the MC or *K* written as $y_{1},\ldots ,y_{150}$, we constructed an algorithm to detect the change points as follows. For t=10,…,150, we raised a change alert if

$$\left|\mathrm{median}\left(y_{t-9},\ldots ,y_{t-5}\right)-\mathrm{median}\left(y_{t-4},\ldots ,y_{t}\right)\right|>\varepsilon \;$$

in the case of MC, and

$$\mathrm{median}\left(y_{t-9},\ldots ,y_{t-5}\right)\neq \mathrm{median}\left(y_{t-4},\ldots ,y_{t}\right)\;$$

in the case of *K*, where ε is the threshold to raise an alert in MC. It should be to some extent large for avoiding too many false alerts and smaller than 1 to find the changes earlier than with monitoring *K*. In this section, we set ε as 0.01 so as not to raise alerts from *t* = 1 to 10 assuming that we know that there are no changes in this period. We calculated the medians instead of the means of the subsequences for robustness. However, to avoid redundant alerts, we neglected them when the difference between *t* and the latest alert was less than 5 even if the conditions were satisfied.

To evaluate the quality of the algorithm, we calculated *Delay* and *False alarm rate* (FAR), defined as

$$\begin{array}{cc} \mathrm{Delay}:= & \min\left(t^{*}-51,50\right),\\ \mathrm{FAR}:= & \frac{\#\{t\in [10,150]|t\notin \mathrm{ACCEPT}\wedge t\in \mathrm{ALERT}\}}{\#\{t\in [10,150]|t\notin \mathrm{ACCEPT}\}},\; \end{array}$$

where $t^{*}$ denotes the first time point in the transaction period when the algorithm generated an alert, ACCEPT denotes the set of time points when alerts can be defined as {t∣∃t−9,…,t∈[51,…,100]}=[51,109], and ALERT denotes the set of time points when the algorithm generates alerts.

#### 6.1.1. Move Gaussian Dataset

The move Gaussian dataset is a set of three-dimensional Gaussian distributions, whose means move gradually in the transaction period. Formally, for each *t*, we generated the data ${\left\{x_{n,t}\right\}}_{n=1}^{1000}$ as follows:

$$x_{n,t}∼\left\{\begin{array}{cc} N\left({x|\mu =\left[0,0,0\right]}^{⊤},\Sigma =I_{3}\right) & (1\leq n\leq 333),\\ N\left({x|\mu =\left[10,0,0\right]}^{⊤},\Sigma =I_{3}\right) & (334\leq n\leq 666),\\ N\left({x|\mu =\left[10+\alpha \left(t\right),0,0\right]}^{⊤},\Sigma =I_{3}\right) & (667\leq n\leq 1000),\; \end{array}\right.$$

where

$$\alpha \left(t\right)=\left\{\begin{array}{cc} 0 & (1\leq t\leq 50),\\ 0.12(t-50) & (51\leq t\leq 100),\\ 6 & (101\leq t\leq 150).\; \end{array}\right.$$

**Figure 6:**
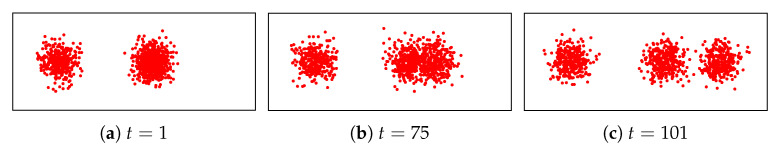
Scatter plots of the first and second dimensions of the data at t=1,75,101 in the move Gaussian dataset.

The first and second dimensions of some data are visualized in Figure 6. In the direction t=1→150, the number of clusters increases from two to three as the two clusters leave; in the direction t=150→1, it decreases from three to two as the two clusters merge.

**Figure 7:**
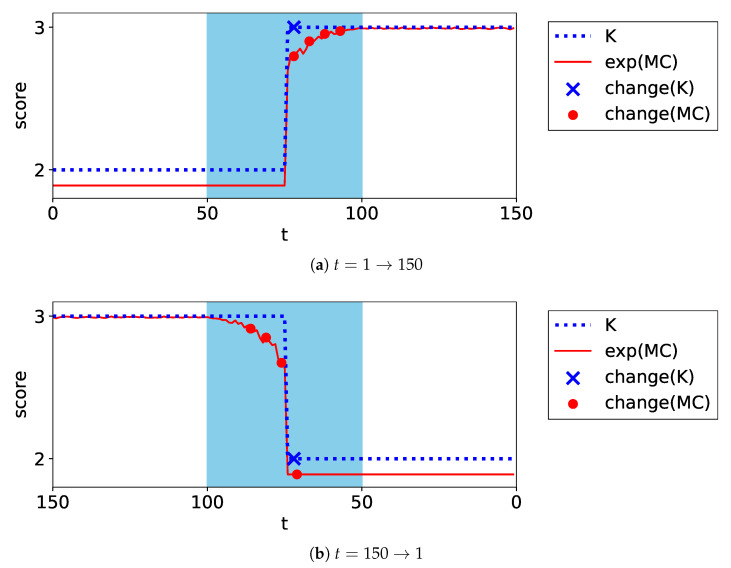
Plots of the exponential of MC and *K* for the move Gaussian dataset. The filled area represents the transaction period. The markers on the plot represent the alerts in each method.

The experiments were performed ten times by randomly generating the datasets; accordingly, the average performance scores were calculated. The differences in the scores between the MC and *K* for each criterion are presented in Table 2; the estimated MC and *K* in one trial are proposed in Figure 7. This figure illustrates the result of BIC as an example.

With respect to the speed to find changes, in every criterion, MC performed as well as *K* in the direction t=1→150; however, it performed significantly better than *K* in the direction t=150→1. The reason for the differing performances is discussed below. In the direction t=1→150, the model selection algorithms underestimated the number of components at the beginning of the transaction period. In such time points, they ignored the overlapping of the two components and considered them as one cluster. Thus, MC, based on such model selection methods, was unable to find the changes earlier than *K*. However, in the direction t=150→1, the overlap between the components was correctly estimated at some time points before *K* changed. In this case, MC changed smoothly according to the overlap and found changes earlier than *K*.

With respect to the accuracy of finding changes, MC performed as well as *K* in terms of FAR. Additionally, it is evident from Figure 7 that MC stably estimated the clustering structures.

#### 6.1.2. Imbalance Gaussian Dataset

The imbalance Gaussian dataset is a set of three-dimensional Gaussian mixture distributions whose balances change gradually in the transaction period. Formally, for each *t*, we generated the data ${\left\{x_{n,t}\right\}}_{n=1}^{1000}$ as follows:

$$x_{n,t}∼\left\{\begin{array}{cc} N\left({x|\mu =\left[0,0,0\right]}^{⊤},\Sigma =I_{3}\right) & (1\leq n\leq 250),\\ N\left({x|\mu =\left[10,0,0\right]}^{⊤},\Sigma =I_{3}\right) & (251\leq n\leq 500),\\ N\left({x|\mu =\left[20,0,0\right]}^{⊤},\Sigma =I_{3}\right) & (501\leq n\leq 750+\alpha (t)),\\ N\left({x|\mu =\left[30,0,0\right]}^{⊤},\Sigma =I_{3}\right) & (751+\alpha (t)\leq n\leq 1000), \end{array}\right.$$

where

$$\alpha \left(t\right)=\left\{\begin{array}{cc} 0 & (1\leq t\leq 50),\\ 5(t-51) & (51\leq t\leq 100),\\ 250 & (101\leq t\leq 150). \end{array}\right.$$

**Figure 8:**
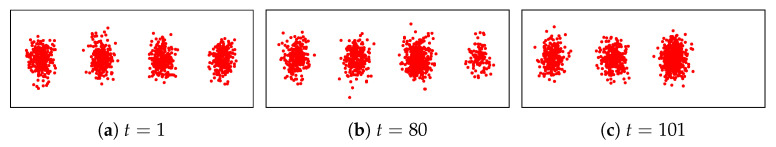
Scatter plots of the first and second dimensions of the data at t=1,80,101 in the imbalance Gaussian dataset.

The first and second dimensions of some data are visualized in Figure 8. In the direction t=1→150, the number of clusters decreases from four to three as the edge cluster disappears. In the direction t=150→1, it increases from three to four as the edge cluster emerges.

**Figure 9:**
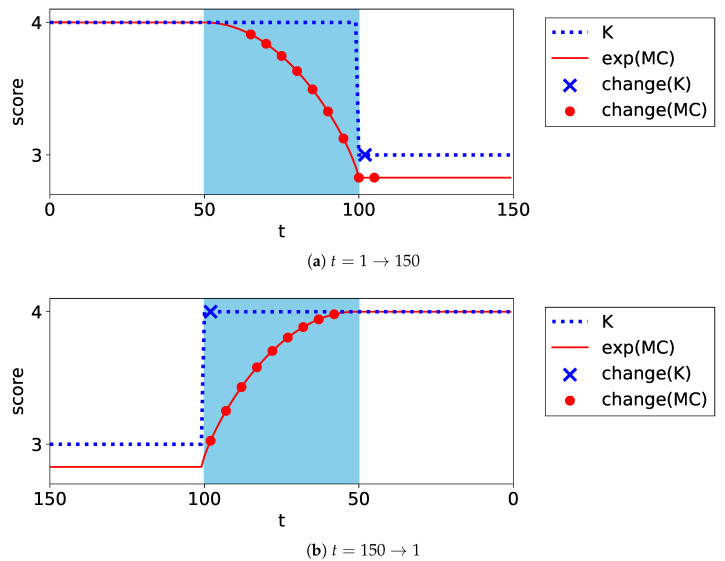
Plots of the exponential of MC and *K* for the imbalance Gaussian dataset. The filled area represents the transaction period. The markers on the plot represent the alerts in each method.

The experiments were performed ten times by randomly generating datasets; accordingly, the average performance scores were calculated. The difference in the scores between the MC and *K* for each criterion are listed in Table 3. The estimated MC and *K* in one trial are plotted in Figure 9. This figure illustrates the result of BIC as an example.

In terms of the speed to find changes, in every model selection method, MC performed significantly better than *K* in the direction t=1→150; however, MC performed as well as *K* in the direction t=150→1. The reason for the differing performances is discussed below. In the transaction period, all model selection methods counted the minor components as independent clusters. Then, in the direction t=1→150, MC changed smoothly according to the imbalance and determined the changes earlier than *K*. In the direction t=150→1, *K* increased significantly early in the transaction period. Then, MC increased along with *K* and determined the changes simultaneously.

In terms of the accuracy of finding changes, MC performed as well as *K* in terms of FAR. Additionally, it is evident from Figure 9 that MC stably estimated the clustering structures.

#### 6.1.3. Scalability

To discuss the scalability for the large datasets, we explored the increase in the computation time for the data size. First, we set the mixture distribution *f* as

$$f\left(x\right)=0.5\times N\left({x|\mu =\left[0,\ldots ,0\right]}^{⊤},\Sigma =I^{d}\right)+0.5\times N\left({x|\mu =\left[1,\ldots ,1\right]}^{⊤},\Sigma =I^{d}\right)\;$$

and sampled *N* points from *f*. Then, we recorded the time to calculate $\left\{\gamma_{k,n}\right\}$ from *f* and calculated the MC from $\left\{\gamma_{k,n}\right\}$. We repeatedly measured the computation times by increasing *N* and *d*. For each *N* and *d*, we measured them ten times and took their averages.

**Figure 10:**
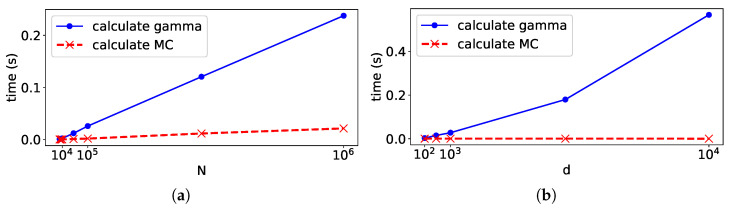
Relationships between the computation time and *N* and *d*. In (**a**), we fixed d=10 and varied *N* from 10 to $10^{6}$. In (**b**), we fixed *N* = 10,000 and varied *d* from 10 to $10^{4}$.

The increase in the computation times is illustrated in Figure 10. In (a), although both computation times increased linearly as *N* grew, calculating MC was faster than calculating $\left\{\gamma_{k,n}\right\}$. In (b), although the time to calculate $\left\{\gamma_{k,n}\right\}$ increased as *d* grew, and the computation time for MC was almost constant because *K* and *N* were not changed. Overall, the cost of computing MC is much smaller than that of computing or estimating $\left\{\gamma_{k,n}\right\}$.

### 6.2. Analysis of Real Data

We analyzed two types of real data named the *beer dataset* and *house dataset*, which are summarized in Table 4. In the following subsections, we discussed the detail of the datasets and results of the experiments.

#### 6.2.1. Beer Dataset

We discuss the results of the beer dataset, obtained from Hakuhodo, Inc. and M-CUBE, Inc. This has also been analyzed in [28,34]. The dataset comprises the records of customer’s beer purchases from November 1st, 2010 to January 31th, 2011. The dataset X is constructed as follows. The time unit is a day. For each day t∈{τ,…,T}, $x_{n,t}\in R^{d}$ denotes the *n*-th customer’s consumption of the beer from time t−τ+1 to *t*, where we set τ=14. The dimension *d* of the vector is 16, which correspond to the consumptions of the following drink:

- beer (A), …, beer (F): beer with brand name A, …, F.
- beer (other): beer with other brands.
- beerlike (A), …, beerlike (H): beer-like drink with brand name A, …, H.
- beerlike (other): beer-like drink with other brands.

**Figure 11:**
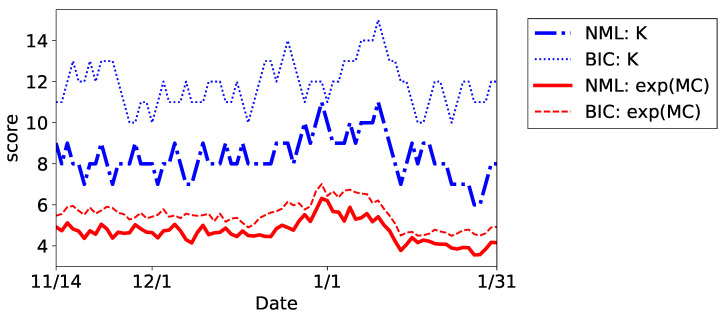
Plots of the sequences of the MC and *K* in the beer dataset.

First, we compare the plots of the estimated MC and *K* in Figure 11. The results of BIC and NML are illustrated as an example. Note that we omit the results of AIC because it chose $K_{\max}$ for ${\hat{K}}_{t}$ at many *t*. In any method, the score was high at the end and beginning of the year, reflecting the increased activities in transactions. However, because the critical changes in the clustering structure and changes due to ineffective components were mixed, the sequence of *K* had a lot of change points; as a result, it was difficult to interpret their meanings. On the other hand, MC identified the clustering structure by discounting the effects of the ineffective components. As a result, the sequence of MC highlighted the significant changes at the end and beginning of the year. It is also worthwhile noting that the differences of the scores between the model selection methods were much smaller in MC than those in *K*; this indicates that both BIC and NML estimates similar clustering structures under the concept of MC even though the number of components differs significantly.

**Figure 12:**
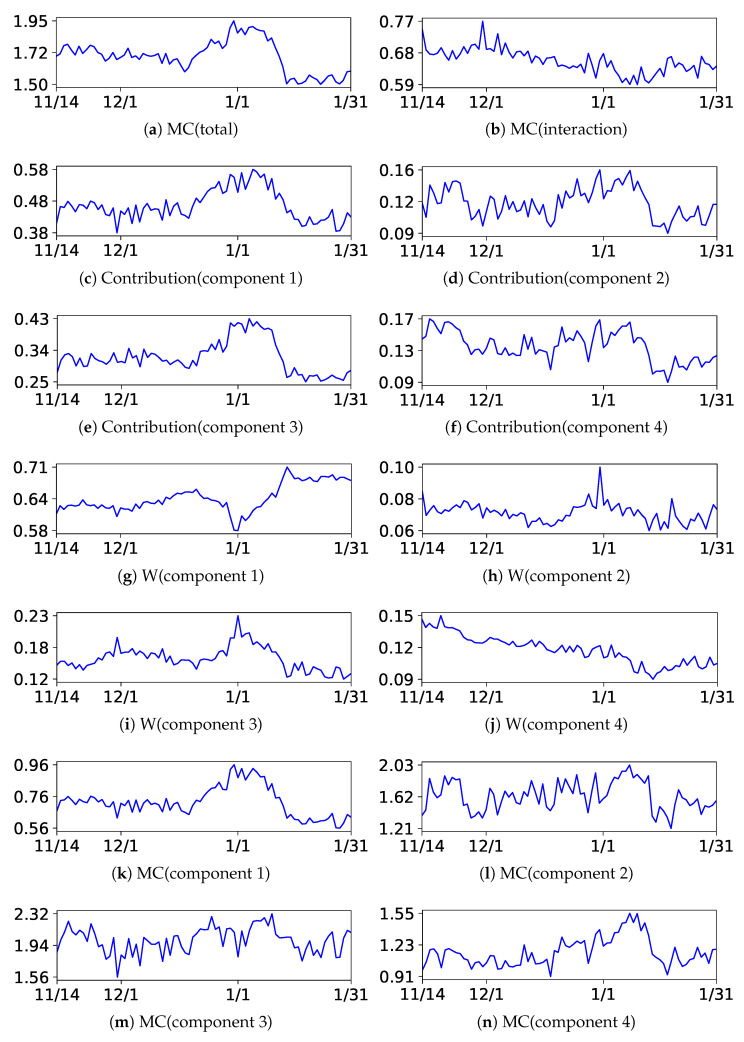
Plots of the decomposition of MC with BIC in the beer Dataset.

**Figure 13:**
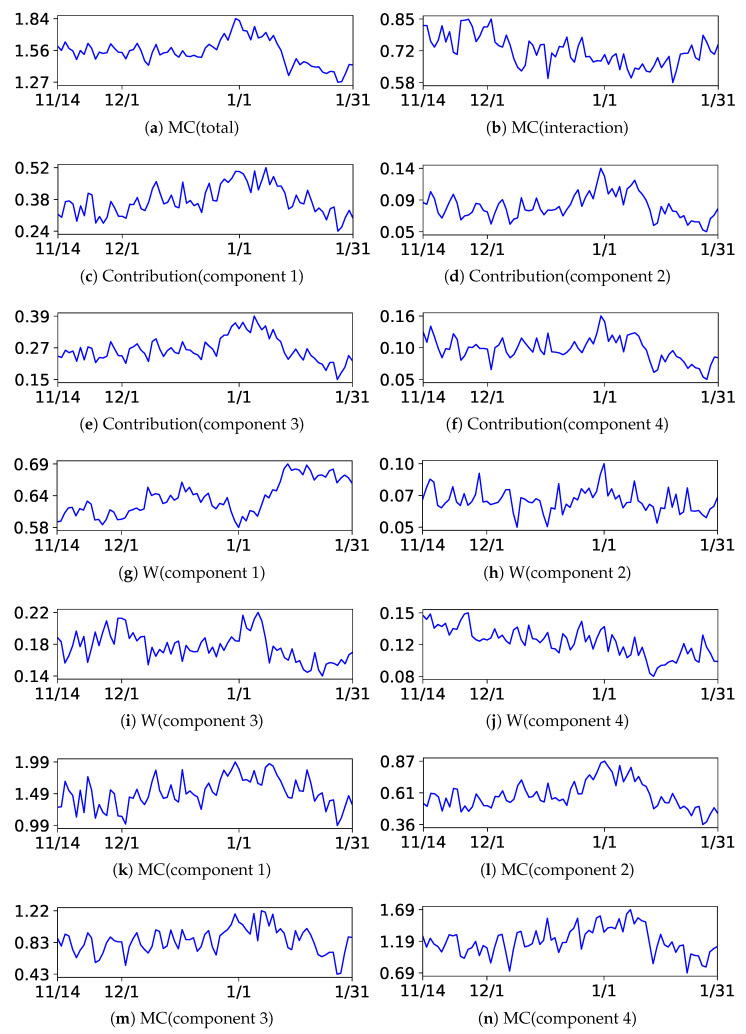
Plots of the decomposition of MC with NML in the beer Dataset.

Next, we discuss the results of the decomposition of MC. We present the results of BIC and NML with L=4 and m=1.5. The centers of the upper components are listed in Table 5 and Table 6, respectively, and the plots of each decomposed value are illustrated in Figure 12 and Figure 13, respectively. The indices of the upper components are manually rearranged so that they correspond with each other; then, it can be observed that the results were also similar to each other. The structures can be extensively evaluated by analyzing the decomposed values. For instance, let us analyze the decomposed values at the end and beginning of the year. As evident from the tables, they had different characteristics. It can be observed from the figures that the contributions increased in all components, indicating that they were related to the increase in MC(total). The weight of the component decreased in cluster 1 and increased in component 2 and 3, indicating that the customers moved from component 1 to component 2 and 3. Additionally, MC(component *l*) increased in all components, indicating that the complexity or diversity increased within them.

#### 6.2.2. House Dataset

We discuss the results of the house dataset, obtained from the UCI Machine Learning Repository [51]. The dataset comprises the records of electricity consumption in a house every five minutes from 16 December 2006 to 26t November 2010. The dataset X is constructed as follows. The time unit is 15 min from 00:00–00:15 to 23:45–24:00. For each *t*, the data ${\left\{x_{n,t}\right\}}_{n=1}^{N}$ denotes the set of the records on the various days included in the *t*-th time unit. The dimension *d* of the vector is 3, which corresponds to the metering of the following three points:

- metering(A): a kitchen.
- metering(B): a laundry room.
- metering(C): a water-heater and an air-conditioner.

**Figure 14:**
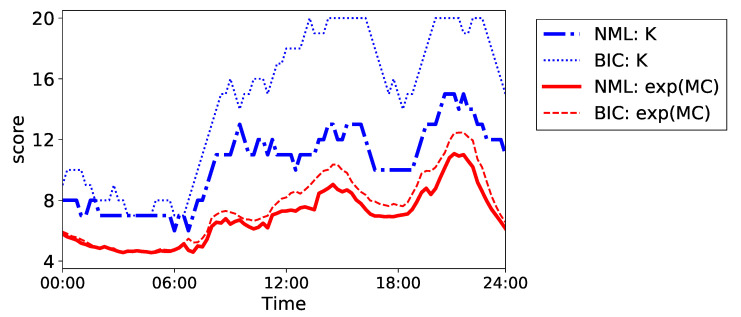
Plots of the sequences of the MC and *K* in the house dataset.

First, we compare the plots of the estimated *K* and the corresponding MC in Figure 14. The results of BIC and NML are illustrated as an example. Note that we omit the results of AIC because it chose $K_{\max}$ for ${\hat{K}}_{t}$ at many *t*. It can be observed from the figure that the MC smoothly connected the discrete changes in *K*; therefore, MC expressed gradual changes in the dataset more effectively than *K*. Additionally, the MCs in BIC and NML were more similar to each other than *K* as well as in the beer dataset. The values of MC started increasing from around 7:00 a.m.; after slight fluctuations, the value reached its peak around 21:00. Therefore, MC seemed to represent the amount of activities in this house.

**Figure 15:**
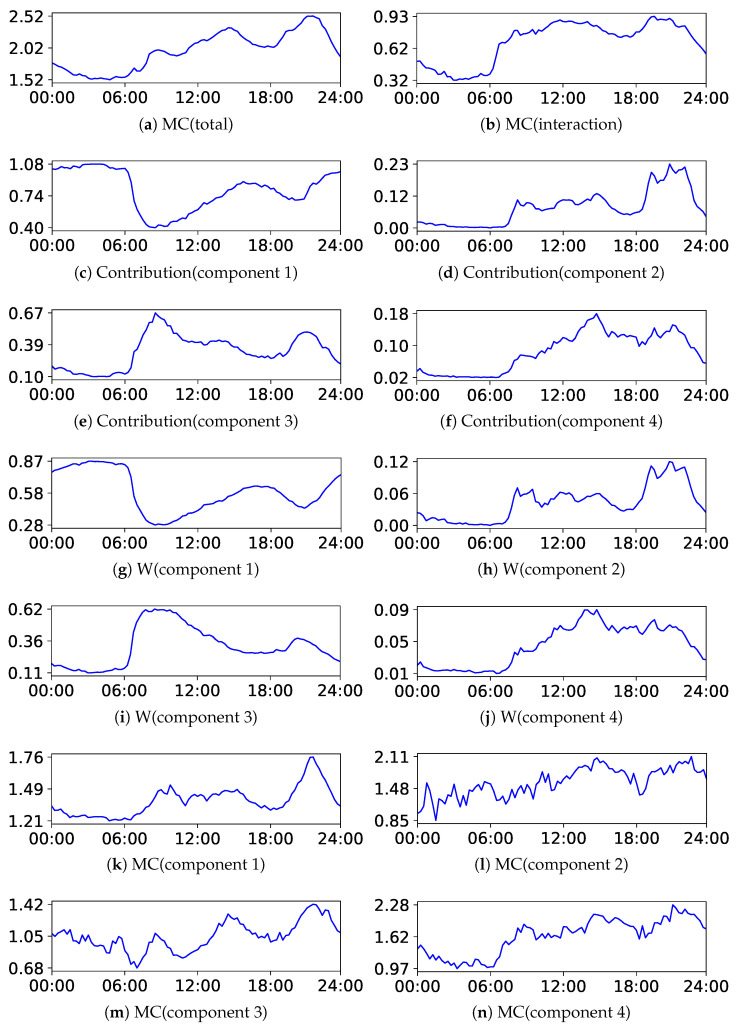
Plots of the decomposition of MC with BIC in the house Dataset.

**Figure 16:**
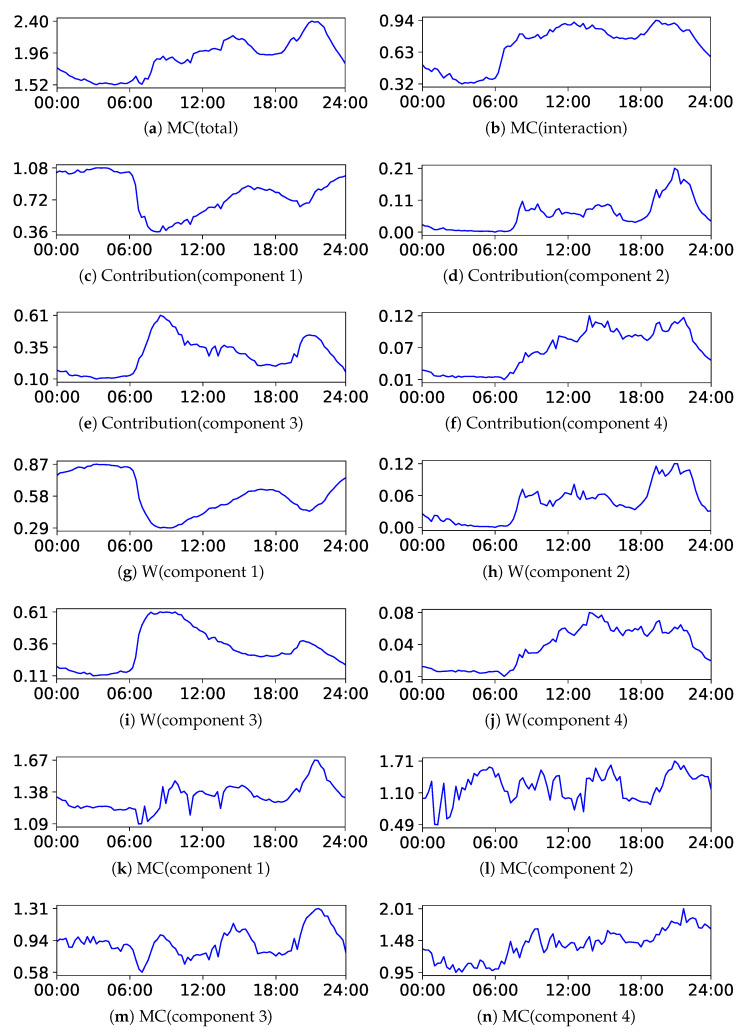
Plots of the decomposition of MC with NML in the house Dataset.

Next, we discuss the results of the decomposition of MC. We present the results of BIC and NML with L=4 and m=1.5. The centers of the upper components are listed in Table 7 and Table 8, respectively, and the plots of each decomposed value are illustrated in Figure 15 and Figure 16, respectively. The indices of the upper components are manually rearranged so that they correspond with each other; then, it can be observed that the results were also similar to each other. The structures can be extensively evaluated by analyzing the decomposed values. For instance, let us analyze the decomposed values in component 3. It can be observed from the tables that the value in metering(C) was specifically high in this component. Looking at the contribution (component 3), there were two peaks around 9:00 and 21:00; it represented the increased activities in this component. However, the proportions of the weight and MC were different. W(component 3) was specifically high at 9:00, indicating that the first half of the peaks was due to the increase in the weight of the component; whereas, MC(component 3) was specifically high at 21:00, indicating that the second half of the peaks was due to the increase in the complexity within the component.

## 7. Conclusions

We proposed the concept of MC to measure the cluster size continuously in the mixture model. We first pointed out that the cluster size might not be equal to the mixture size when the mixture model had overlap or weight bias; then, we introduced MC as an extended concept of the cluster size considering the effects of them. We also presented methods to decompose the MC according to the mixture hierarchies, which helped us in extensively analyzing the substructures. Subsequently, we implemented the MC and its decomposition to the gradual clustering change detection problems. We conducted experiments to verify that the MC effectively elucidates the clustering changes. In the artificial data experiments, MC found the clustering changes significantly earlier in the case where the overlap or weight bias was correctly estimated. In the real data experiments, MC expressed the gradual changes better than *K* because it discerned the significant and insignificant changes and smoothly connected the discrete changes in *K*. We also found that the MC took similar values for each model selection method; it indicates that the estimated clustering structures are alike under the concept of MC. Moreover, its decomposition enabled us to evaluate the contents of changes.

Issues of the MC will be tackled in future study. For example, it does not capture the clustering structure well when the number of the components is underestimated; thus, we need to explore the model selection methods that are more compatible with MC. Also, we further need to study its theoretical aspects, such as convergence and methods for approximating the mutual information. Furthremore, we need to consider extending the concept of MC into other clustering approaches, e.g., considering co-clustering by relating non-diagonal blocks in co-clustering and cluster overlaps in finite mixture models.

## Figures and Tables

**Table 1:** Quantities in the example of the decomposition.

	Component 1	Component 2
MC (total)	1.076	
MC (interaction)	0.643	
Contribution (component *l*)	0.277	0.157
W (component *l*)	0.496	0.504
MC (component *l*)	0.558	0.311

**Table 2:** Difference in the average performance score between MC and *K* for the move Gaussian dataset.

	(Score of MC) − (Score of *K*)			
	t=1→150		t=150→1	
Criterion	Delay	FAR	Delay	Far
AIC	0.0	0.000	−20.6	0.000
AIC+comp	0.0	0.000	−10.9	0.000
BIC	0.0	0.000	−17.5	0.000
BIC+comp	0.0	0.000	−8.9	0.000
NML	0.0	0.000	−7.9	0.000
DNML	0.0	0.000	−7.7	0.000

**Table 3:** Differences in the average performance score between MC and *K* for the imbalance Gaussian dataset.

	(Score of MC) − (Score of *K*)			
	t=1→150		t=150→1	
Criterion	Delay	FAR	Delay	Far
AIC	−30.2	0.010	−4.6	0.000
AIC+comp	−34.0	0.000	0.0	0.000
BIC	−34.0	0.000	0.0	0.000
BIC+comp	−34.0	0.000	0.0	0.000
NML	−34.0	0.000	0.0	0.000
DNML	−34.0	0.000	0.0	0.000

**Table 4:** Summary of the dataset.

Dataset	*T*	$N_{t}$	*d*	Description
beer	92	3185	16	purchase data of beer.
house	96	4326	3	electricity consumption data in a house.

**Table 5:** Centers of the upper components estimated by BIC in the beer dataset. For each dimension, the maximum value is denoted in boldface.

	Component 1	Component 2	Component 3	Component 4
beer(A)	0.09	0.44	**1.93**	0.16
beer(B)	0.07	0.23	**0.96**	0.06
beer(C)	0.07	0.20	**0.83**	0.07
beer(D)	0.05	0.20	**0.58**	0.05
beer(E)	0.03	0.06	**0.35**	0.03
beer(F)	0.03	0.06	**0.35**	0.02
beer(other)	0.04	0.12	**0.69**	0.10
beerlike(A)	0.02	**5.85**	0.23	0.07
beerlike(B)	0.09	0.57	**0.80**	0.21
beerlike(C)	0.10	0.63	**0.83**	0.22
beerlike(D)	0.07	0.40	**0.57**	0.18
beerlike(E)	0.04	0.12	**0.51**	0.06
beerlike(F)	0.04	0.20	**0.34**	0.13
beerlike(G)	0.05	0.10	**0.40**	0.06
beerlike(H)	0.03	0.09	**0.26**	0.04
beerlike(other)	0.09	1.27	1.11	**6.78**

**Table 6:** Centers of the upper components estimated by NML in the beer dataset. For each dimension, the maximum value is denoted in boldface.

	Component 1	Component 2	Component 3	Component 4
beer(A)	0.08	0.48	**1.90**	0.12
beer(B)	0.04	0.30	**1.04**	0.07
beer(C)	0.05	0.20	**0.95**	0.04
beer(D)	0.04	0.19	**0.64**	0.09
beer(E)	0.02	0.06	**0.38**	0.02
beer(F)	0.02	0.07	**0.40**	0.01
beer(other)	0.03	0.11	**0.68**	0.19
beerlike(A)	0.02	**5.79**	0.21	0.07
beerlike(B)	0.10	0.52	**0.73**	0.18
beerlike(C)	0.11	0.61	**0.70**	0.21
beerlike(D)	0.06	0.49	**0.52**	0.24
beerlike(E)	0.04	0.12	**0.47**	0.07
beerlike(F)	0.04	0.18	**0.30**	0.24
beerlike(G)	0.04	0.11	**0.44**	0.07
beerlike(H)	0.02	0.10	**0.23**	0.09
beerlike(other)	0.08	1.42	1.08	**6.54**

**Table 7:** Centers of the upper components estimated by BIC in the house dataset. For each dimension, the maximum value is denoted in boldface.

	Component 1	Component 2	Component 3	Component 4
metering(A)	0.04	**4.47**	0.13	0.41
metering(B)	0.53	0.89	0.56	**4.40**
metering(C)	0.75	3.34	**4.37**	2.96

**Table 8:** Centers of the upper components estimated by NML in the house dataset. For each dimension, the maximum value is denoted in boldface.

	Component 1	Component 2	Component 3	Component 4
metering(A)	0.04	**4.24**	0.11	0.35
metering(B)	0.53	1.00	0.57	**4.48**
metering(C)	0.76	3.37	**4.38**	2.93

## Data Availability

Not applicable.

## Appendix A. Proof of the Basic Properties

We present a proof of Propositions 1–4.

### Appendix A.1. Proof of Proposition 1

We can directly calculate as follows:

$$\begin{array}{cc} \; & \;\mathrm{MC}\left({\left\{\gamma_{k}\left(x_{n}\right)\right\}}_{k,n};{\left\{w_{n}\right\}}_{n}\right)\\ = & \;-\sum_{k=1}^{K}\left(\frac{\sum_{n}w_{n}\gamma_{k}}{\sum_{n^{\prime }}w_{n^{\prime }}}\right)\log\left(\frac{\sum_{n}w_{n}\gamma_{k}}{\sum_{n^{\prime }}w_{n^{\prime }}}\right)+\frac{1}{\sum_{n^{\prime }}w_{n^{\prime }}}\sum_{n=1}^{N}w_{n}\sum_{k=1}^{K}\gamma_{k}\log\gamma_{k}\\ = & \;-\sum_{k=1}^{K}\gamma_{k}\log\gamma_{k}+\sum_{k=1}^{K}\gamma_{k}\log\gamma_{k}\\ = & \;0. \end{array}$$

### Appendix A.2. Proof of Proposition 2

Because xlogx=0 when x=0 or 1, $\tilde{H}\left(Z|X\right)$ becomes 0 when the components are entirely separate. Thus, the proposition hols.

### Appendix A.3. Proof of Proposition 3

It is immediate that

$$\mathrm{MC}\left({\left\{\gamma_{k}\left(x_{n}\right)\right\}}_{k,n};{\left\{w_{n}\right\}}_{n}\right)=\tilde{H}\left(Z\right)-\tilde{H}\left(Z|X\right)\overset{\left(a\right)}{\leq }\tilde{H}\left(Z\right)\overset{\left(b\right)}{\leq }\log K.\;$$

The equality of (a) holds only if the components are entirely separate and the equality of (b) holds only if the components are balanced. Thus, MC equals logK only if the components are entirely separate and balanced.

Also, by applying the Jensen’s inequality to x↦−xlogx, we obtain that

$$-\sum_{k=1}^{K}\left(\frac{\sum_{n}w_{n}\gamma_{k}\left(x_{n}\right)}{\sum_{n^{\prime }}w_{n^{\prime }}}\right)\log\left(\frac{\sum_{n}w_{n}\gamma_{k}\left(x_{n}\right)}{\sum_{n^{\prime }}w_{n^{\prime }}}\right)\geq -\sum_{k=1}^{K}\frac{1}{\sum_{n^{\prime }}w_{n^{\prime }}}\sum_{n=1}^{N}w_{n}\gamma_{k}\left(x_{n}\right)\log\gamma_{k}\left(x_{n}\right),\;$$

which is equivalent to that $\mathrm{MC}({\left\{\gamma_{k}\left(x_{n}\right)\right\}}_{k,n},{\left\{w_{n}\right\}}_{n})\geq 0$. The equality holds only if the components entirely overlap.

### Appendix A.4. Proof of Proposition 4

By applying Theorem 1 to partition $I_{1}\cup \ldots \cup I_{L}$ into each set, we can calculate that

$$\begin{array}{cc} \; & \;\mathrm{MC}\left({\left\{\gamma_{k}\left(x_{n}\right)\right\}}_{k,n};{\left\{w_{n}\right\}}_{n}\right)\\ = & \;\mathrm{MC}\left({\left\{\gamma_{k}\left(x_{n}\right)\right\}}_{k\in I_{1}\cup \ldots \cup I_{L},n};{\left\{w_{n}\right\}}_{n}\right)\\ = & \;\mathrm{MC}\left({\left\{\sum_{k\in I_{l}}\gamma_{k}\left(x_{n}\right)\right\}}_{l=1,\ldots ,L,n};{\left\{w_{n}\right\}}_{n}\right)\\ \; & \;\;+\sum_{l=1}^{L}\left(\sum_{k\in I_{l}}{\tilde{\rho }}_{l}\right)\mathrm{MC}\left({\left\{\frac{\gamma_{k}\left(x_{n}\right)}{\sum_{k^{\prime }\in I_{l}}\gamma_{k^{\prime }}\left(x_{n}\right)}\right\}}_{k\in I_{l},n};{\left\{w_{n}\sum_{k\in I_{l}}\gamma_{k}\left(x_{n}\right)\right\}}_{n}\right)\\ = & \;\mathrm{MC}\left({\left\{\gamma_{k}^{0}\left(x_{n}\right)\right\}}_{k,n};{\left\{w_{n}\right\}}_{n}\right)\\ \; & \;\;+\sum_{l=1}^{L}\left(\sum_{k\in I_{l}}{\tilde{\rho }}_{l}\right)\mathrm{MC}\left({\left\{\frac{\rho_{k}}{\sum_{k^{\prime }\in I_{l}}\rho_{k^{\prime }}}\right\}}_{k\in I_{l},n};{\left\{w_{n}\sum_{k\in I_{l}}\gamma_{k}\left(x_{n}\right)\right\}}_{n}\right)\\ = & \;\mathrm{MC}\left({\left\{\gamma_{k}^{0}\left(x_{n}\right)\right\}}_{k,n};{\left\{w_{n}\right\}}_{n}\right)\;\left(∵\;\mathrm{Proposition}\;\right). \end{array}$$

## Appendix B. Proof of the Decomposition Property

We present a proof of Theorem Table 1. Let

$$\begin{array}{c} {\tilde{\rho }}_{k}^{\left(l\right)}=\frac{\sum_{n}w_{n}^{\left(l\right)}\gamma_{k}^{\left(l\right)}\left(x_{n}\right)}{\sum_{n^{\prime }}w_{n^{\prime }}^{\left(l\right)}}=\frac{\sum_{n}w_{n}Q_{k}^{\left(l\right)}\gamma_{k}\left(x_{n}\right)}{\sum_{n^{\prime }}w_{n^{\prime }}\sum_{k^{\prime }}Q_{k^{\prime }}^{\left(l\right)}\gamma_{k^{\prime }}\left(x_{n^{\prime }}\right)}.\; \end{array}$$

Then, we can calculate as

$$\begin{array}{cc} \; & \;\mathrm{MC}\left({\left\{\gamma_{k}\left(x_{n}\right)\right\}}_{k,n};{\left\{w_{n}\right\}}_{n}\right)-\mathrm{MC}\left({\left\{\sum_{k=1}^{K}Q_{k}^{\left(l\right)}\gamma_{k}\left(x_{n}\right)\right\}}_{l,n};{\left\{w_{n}\right\}}_{n}\right)\\ = & \;\sum_{n=1}^{N}\sum_{k=1}^{K}\sum_{l=1}^{L}\frac{w_{n}Q_{k}^{\left(l\right)}\gamma_{k}\left(x_{n}\right)}{\sum_{n^{\prime }}w_{n^{\prime }}}\log\left(\frac{\gamma_{k}\left(x_{n}\right)}{\frac{\sum_{n^{\prime }}w_{n^{\prime }}\gamma_{k}\left(x_{n^{\prime }}\right)}{\sum_{n^{\prime }}w_{n^{\prime }}}}\cdot \frac{\frac{\sum_{n^{\prime }}w_{n^{\prime }}\sum_{k^{\prime }}Q_{k^{\prime }}^{\left(l\right)}\gamma_{k^{\prime }}\left(x_{n^{\prime }}\right)}{\sum_{n^{\prime }}w_{n^{\prime }}}}{\sum_{k^{\prime }}Q_{k^{\prime }}^{\left(l\right)}\gamma_{k^{\prime }}\left(x_{n}\right)}\right)\\ = & \;\sum_{n=1}^{N}\sum_{k=1}^{K}\sum_{l=1}^{L}\frac{w_{n}Q_{k}^{\left(l\right)}\gamma_{k}\left(x_{n}\right)}{\sum_{n^{\prime }}w_{n^{\prime }}}\log\left(\frac{Q_{k}^{\left(l\right)}\gamma_{k}\left(x_{n}\right)}{\sum_{k^{\prime }}Q_{k^{\prime }}^{\left(l\right)}\gamma_{k^{\prime }}\left(x_{n}\right)}\cdot \frac{\sum_{n^{\prime }}w_{n^{\prime }}\sum_{k^{\prime }}Q_{k^{\prime }}^{\left(l\right)}\gamma_{k^{\prime }}\left(x_{n^{\prime }}\right)}{\sum_{n^{\prime }}w_{n^{\prime }}Q_{k}^{\left(l\right)}\gamma_{k}\left(x_{n^{\prime }}\right)}\right)\\ = & \;\sum_{n=1}^{N}\sum_{k=1}^{K}\sum_{l=1}^{L}W_{l}\cdot \frac{w_{n}Q_{k}^{\left(l\right)}\gamma_{k}\left(x_{n}\right)}{\sum_{n^{\prime }}w_{n^{\prime }}\sum_{k^{\prime }}Q_{k^{\prime }}^{\left(l\right)}\gamma_{k^{\prime }}\left(x_{n^{\prime }}\right)}\log\left(\frac{\gamma_{k}^{\left(l\right)}\left(x_{n}\right)}{{\tilde{\rho }}_{k}^{\left(l\right)}}\right)\\ = & \;\sum_{n=1}^{N}\sum_{k=1}^{K}\sum_{l=1}^{L}W_{l}\cdot \frac{w_{n}^{\left(l\right)}\gamma_{k}^{\left(l\right)}\left(x_{n}\right)}{\sum_{n^{\prime }}w_{n^{\prime }}^{\left(l\right)}}\log\left(\frac{\gamma_{k}^{\left(l\right)}\left(x_{n}\right)}{{\tilde{\rho }}_{k}^{\left(l\right)}}\right)\\ = & \;\sum_{l=1}^{L}W_{l}\cdot \left({\left\{\gamma_{k}^{\left(l\right)}\left(x_{n}\right)\right\}}_{k,n};{\left\{w_{n}^{\left(l\right)}\right\}}_{n}\right). \end{array}$$

## Appendix C. Proof of the Consistency

We present a proof of Theorem 2. We use the following notations for convenience:

$$\begin{array}{cc} \left[\gamma_{1}\left(x_{n}\right)\left|\ldots \right|\gamma_{K}\left(x_{n}\right)\right]:= & {\left\{\gamma_{k}\left(x_{n}\right)\right\}}_{k,n},\\ I_{0}:= & I_{1}\cup \ldots \cup I_{K^{☆}}.\; \end{array}$$

First, applying Theorem 1 repeatedly, we decompose the entire MC as follows:

(A1) $$\begin{array}{cc} \; & \;\mathrm{MC}\left({\left\{\gamma_{k}\left(x_{n}\right)\right\}}_{k,n}\right)\\ = & \;\mathrm{MC}\left(\left[\sum_{k\in I_{0}}\gamma_{k}\left(x_{n}\right)\;|\;\sum_{k\in I_{\infty }}\gamma_{k}\left(x_{n}\right)\right]\right)\\ \; & \;\;+{\tilde{\rho }}_{\infty }\mathrm{MC}\left({\left\{\frac{\gamma_{k}\left(x_{n}\right)}{\sum_{k^{\prime }\in I_{\infty }}\gamma_{k^{\prime }}\left(x_{n}\right)}\right\}}_{k\in I_{\infty },n};\left\{\sum_{k\in I_{\infty }}\gamma_{k}\left(x_{n}\right)\right\}\right)\\ \; & \;\;+\left(1-{\tilde{\rho }}_{\infty }\right)\mathrm{MC}\left({\left\{\frac{\gamma_{k}\left(x_{n}\right)}{\sum_{k^{\prime }\in I_{0}}\gamma_{k^{\prime }}\left(x_{n}\right)}\right\}}_{k\in I_{0},n};\left\{\sum_{k\in I_{0}}\gamma_{k}\left(x_{n}\right)\right\}\right)\\ = & \;\mathrm{MC}\left(\left[\sum_{k\in I_{0}}\gamma_{k}\left(x_{n}\right)\;|\;\sum_{k\in I_{\infty }}\gamma_{k}\left(x_{n}\right)\right]\right)\\ \; & \;\;+{\tilde{\rho }}_{\infty }\;\mathrm{MC}\left({\left\{\frac{\gamma_{k}\left(x_{n}\right)}{\sum_{k^{\prime }\in I_{\infty }}\gamma_{k^{\prime }}\left(x_{n}\right)}\right\}}_{k\in I_{\infty },n};\left\{\sum_{k\in I_{\infty }}\gamma_{k}\left(x_{n}\right)\right\}\right)\\ \; & \;\;+\left(1-{\tilde{\rho }}_{\infty }\right)\mathrm{MC}\left({\left\{\frac{\sum_{k\in I_{l}}\gamma_{k}\left(x_{n}\right)}{\sum_{k^{\prime }\in I_{0}}\gamma_{k^{\prime }}\left(x_{n}\right)}\right\}}_{l=1,\ldots ,K^{☆}};\left\{\sum_{k\in I_{0}}\gamma_{k}\left(x_{n}\right)\right\}\right)\\ \; & \;\;+\sum_{l=1}^{K^{☆}}\left(\sum_{k\in I_{l}}{\tilde{\rho }}_{k}\right)\mathrm{MC}\left({\left\{\frac{\gamma_{k}\left(x_{n}\right)}{\sum_{k^{\prime }\in I_{l}}\gamma_{k^{\prime }}\left(x_{n}\right)}\right\}}_{k\in I_{l}};\left\{\sum_{k\in I_{l}}\gamma_{k}\left(x_{n}\right)\right\}\right)\\ = & \;\mathrm{MC}\left(\left[\frac{\sum_{l=1}^{K^{☆}}r_{l}h_{l}\left(x_{n}\right)}{f\left(x_{n}\right)}\;|\;\frac{\sum_{k\in I_{\infty }}\rho_{k}g_{k}\left(x_{n}\right)}{f\left(x_{n}\right)}\right]\right)\\ \; & \;\;+{\tilde{\rho }}_{\infty }\mathrm{MC}\left({\left\{\frac{\rho_{k}\left(x_{n}\right)}{\sum_{k^{\prime }\in I_{\infty }}\rho_{k^{\prime }}\left(x_{n}\right)}\right\}}_{k\in I_{\infty }};\left\{\sum_{k\in I_{\infty }}\gamma_{k}\left(x_{n}\right)\right\}\right)\\ \; & \;\;+\left(1-{\tilde{\rho }}_{\infty }\right)\mathrm{MC}\left({\left\{\frac{r_{l}h_{l}\left(x_{n}\right)}{\sum_{l^{\prime }=1}^{K^{☆}}r_{l^{\prime }}h_{l^{\prime }}\left(x_{n}\right)}\right\}}_{l=1,\ldots ,K^{☆}};\left\{\sum_{k\in I_{0}}\gamma_{k}\left(x_{n}\right)\right\}\right)\\ \; & \;\;+\sum_{l=1}^{K^{☆}}\left(\sum_{k\in I_{l}}{\tilde{\rho }}_{k}\right)\mathrm{MC}\left({\left\{\frac{s_{k}g_{k}\left(x_{n}\right)}{h_{l}\left(x_{n}\right)}\right\}}_{k\in I_{l}};\left\{\sum_{k\in I_{l}}\gamma_{k}\left(x_{n}\right)\right\}\right). \end{array}$$

The terms in (A1) correspond to the four quantities (a), …, (d) referred to in Section 4.3. Then, it is sufficient to show that

$$\begin{array}{cc} \mathrm{MC}\left(\left[\frac{\sum_{l=1}^{K^{☆}}r_{l}h_{l}\left(x_{n}\right)}{f\left(x_{n}\right)}\;|\;\frac{\sum_{k\in I_{\infty }}\rho_{k}g_{k}\left(x_{n}\right)}{f\left(x_{n}\right)}\right]\right)\; & \to 0,\\ {\tilde{\rho }}_{\infty }\mathrm{MC}\left({\left\{\frac{\rho_{k}\left(x_{n}\right)}{\sum_{k^{\prime }\in I_{\infty }}\rho_{k^{\prime }}\left(x_{n}\right)}\right\}}_{k\in I_{\infty }};\left\{\sum_{k\in I_{\infty }}\gamma_{k}\left(x_{n}\right)\right\}\right)\; & \to 0,\\ \left(1-{\tilde{\rho }}_{\infty }\right)\mathrm{MC}\left({\left\{\frac{r_{l}h_{l}\left(x_{n}\right)}{\sum_{l^{\prime }=1}^{K^{☆}}r_{l^{\prime }}h_{l^{\prime }}\left(x_{n}\right)}\right\}}_{l=1,\ldots ,K^{☆}};\left\{\sum_{k\in I_{0}}\gamma_{k}\left(x_{n}\right)\right\}\right)\; & \to \mathrm{MC}\left({\left\{\gamma_{k}^{☆}\left(x_{n}\right)\right\}}_{k,n}\right),\\ \sum_{l=1}^{K^{☆}}\left(\sum_{k\in I_{l}}{\tilde{\rho }}_{k}\right)\mathrm{MC}\left({\left\{\frac{s_{k}g_{k}\left(x_{n}\right)}{h_{l}\left(x_{n}\right)}\right\}}_{k\in I_{l}};\left\{\sum_{k\in I_{l}}\gamma_{k}\left(x_{n}\right)\right\}\right)\; & \to 0. \end{array}$$

Step 1

First, we show that

$$\mathrm{MC}\left(\left[\frac{\sum_{l=1}^{K^{☆}}r_{l}h_{l}\left(x_{n}\right)}{f\left(x_{n}\right)}\;|\;\frac{\sum_{k\in I_{\infty }}\rho_{k}g_{k}\left(x_{n}\right)}{f\left(x_{n}\right)}\right]\right)=O_{P}\left({\tilde{\rho }}_{\infty }\left(-\log{\tilde{\rho }}_{\infty }\right)\right).$$

Using Proposition 3, it is easily shown as follows:

$$\begin{array}{cc} \mathrm{MC}\left(\left[\frac{\sum_{l=1}^{K^{☆}}r_{l}h_{l}\left(x_{n}\right)}{f\left(x_{n}\right)}\;|\;\frac{\sum_{k\in I_{\infty }}\rho_{k}g_{k}\left(x_{n}\right)}{f\left(x_{n}\right)}\right]\right)\; & \leq -{\tilde{\rho }}_{\infty }\log{\tilde{\rho }}_{\infty }-\left(1-{\tilde{\rho }}_{\infty }\right)\log\left(1-{\tilde{\rho }}_{\infty }\right)\\ \; & =O_{P}\left({\tilde{\rho }}_{\infty }\left(-\log{\tilde{\rho }}_{\infty }\right)\right). \end{array}$$

Step 2

Second, we show that

$${\tilde{\rho }}_{\infty }\mathrm{MC}\left({\left\{\frac{\rho_{k}\left(x_{n}\right)}{\sum_{k^{\prime }\in I_{\infty }}\rho_{k^{\prime }}\left(x_{n}\right)}\right\}}_{k\in I_{\infty }};\left\{\sum_{k\in I_{\infty }}\gamma_{k}\left(x_{n}\right)\right\}\right)=O_{P}\left({\tilde{\rho }}_{\infty }\right).$$

It is also evident from Proposition 3:

$$\begin{array}{cc} {\tilde{\rho }}_{\infty }\mathrm{MC}\left({\left\{\frac{\rho_{k}\left(x_{n}\right)}{\sum_{k^{\prime }\in I_{\infty }}\rho_{k^{\prime }}\left(x_{n}\right)}\right\}}_{k\in I_{\infty }};\left\{\sum_{k\in I_{\infty }}\gamma_{k}\left(x_{n}\right)\right\}\right)\; & \leq {\tilde{\rho }}_{\infty }\;\log\left(K-i_{K^{☆}}\right)\\ \; & =O_{P}\left({\tilde{\rho }}_{\infty }\right). \end{array}$$

Step 3

Third, we show that

$$\begin{array}{cc} \; & \;\left(1-{\tilde{\rho }}_{\infty }\right)\mathrm{MC}\left({\left\{\frac{r_{l}h_{l}\left(x_{n}\right)}{\sum_{l^{\prime }=1}^{K^{☆}}r_{l^{\prime }}h_{l^{\prime }}\left(x_{n}\right)}\right\}}_{l=1,\ldots ,K^{☆}};\left\{\sum_{k\in I_{0}}\gamma_{k}\left(x_{n}\right)\right\}\right)\\ = & \;\mathrm{MC}\left({\left\{\gamma_{k}^{☆}\left(x_{n}\right)\right\}}_{k,n}\right)+O_{P}\left(∥\phi -\phi^{☆}∥+\sum_{l=1}^{K^{☆}}\left|{\tilde{r}}_{l}-\rho_{l}^{☆}\right|+{\tilde{\rho }}_{\infty }\right). \end{array}$$

To this end, we further decompose the left hand as

$$\begin{array}{cc} \; & \;\left(1-{\tilde{\rho }}_{\infty }\right)\mathrm{MC}\left({\left\{\frac{r_{l}h_{l}\left(x_{n}\right)}{\sum_{l^{\prime }=1}^{K^{☆}}r_{l^{\prime }}h_{l^{\prime }}\left(x_{n}\right)}\right\}}_{\;l=1,\ldots ,K^{☆}};\left\{\sum_{k\in I_{0}}\gamma_{k}\left(x_{n}\right)\right\}\right)\\ = & \;-\left(1-{\tilde{\rho }}_{\infty }\right)\sum_{l=1}^{K^{☆}}\frac{{\tilde{r}}_{l}}{1-{\tilde{\rho }}_{\infty }}\log\left(\frac{{\tilde{r}}_{l}}{1-{\tilde{\rho }}_{\infty }}\right)\\ \; & \;\;+\frac{1}{N}\sum_{n=1}^{N}\sum_{l=1}^{K^{☆}}\left(\sum_{k\in I_{0}}\gamma_{k}\left(x_{n}\right)\right)\frac{r_{l}h_{l}\left(x_{n}\right)}{\sum_{l^{\prime }=1}^{K^{☆}}r_{l^{\prime }}h_{l^{\prime }}\left(x_{n}\right)}\log\left(\frac{r_{l}h_{l}\left(x_{n}\right)}{\sum_{l^{\prime }=1}^{K^{☆}}r_{l^{\prime }}h_{l^{\prime }}\left(x_{n}\right)}\right); \end{array}$$

they correspond to the unconditional and conditional entropy of the latent variables, respectively. On the other hand, the true MC is defined as

$$-\sum_{l=1}^{K^{☆}}{\tilde{\rho }}_{l}^{☆}\log{\tilde{\rho }}_{l}^{☆}+\frac{1}{N}\sum_{n=1}^{N}\sum_{l=1}^{K^{☆}}\gamma_{l}^{☆}\left(x_{n}\right)\log\gamma_{l}^{☆}\left(x_{n}\right).$$

Then, it is sufficient to show that

$$\begin{array}{c} -\left(1-{\tilde{\rho }}_{\infty }\right)\sum_{l=1}^{K^{☆}}\frac{{\tilde{r}}_{l}}{1-{\tilde{\rho }}_{\infty }}\log\left(\frac{{\tilde{r}}_{l}}{1-{\tilde{\rho }}_{\infty }}\right)\to -\sum_{l=1}^{K^{☆}}{\tilde{\rho }}_{l}^{☆}\log{\tilde{\rho }}_{l}^{☆},\\ \begin{array}{c} \frac{1}{N}\sum_{n=1}^{N}\sum_{l=1}^{K^{☆}}\left(\sum_{k\in I_{0}}\gamma_{k}\left(x_{n}\right)\right)\frac{r_{l}h_{l}\left(x_{n}\right)}{\sum_{l^{\prime }=1}^{K^{☆}}r_{l^{\prime }}h_{l^{\prime }}\left(x_{n}\right)}\log\left(\frac{r_{l}h_{l}\left(x_{n}\right)}{\sum_{l^{\prime }=1}^{K^{☆}}r_{l^{\prime }}h_{l^{\prime }}\left(x_{n}\right)}\right)\\ \to \frac{1}{N}\sum_{n=1}^{N}\sum_{l=1}^{K^{☆}}\gamma_{l}^{☆}\left(x_{n}\right)\log\gamma_{l}^{☆}\left(x_{n}\right).\; \end{array} \end{array}$$

First, we show that

$$\begin{array}{cc} \; & \;-\left(1-{\tilde{\rho }}_{\infty }\right)\sum_{l=1}^{K^{☆}}\frac{{\tilde{r}}_{l}}{1-{\tilde{\rho }}_{\infty }}\log\left(\frac{{\tilde{r}}_{l}}{1-{\tilde{\rho }}_{\infty }}\right)\\ = & \;-\sum_{l=1}^{K^{☆}}{\tilde{\rho }}_{l}^{☆}\log{\tilde{\rho }}_{l}^{☆}+O_{P}\left(\sum_{l=1}^{K^{☆}}\left|{\tilde{r}}_{l}-\rho_{l}^{☆}\right|+{\tilde{\rho }}_{\infty }+\frac{1}{\sqrt{N}}\right).\; \end{array}$$

Indeed, by the mean-value theorem, there exist $r_{1}^{m},\ldots ,r_{K^{☆}}^{m}$ between ${\tilde{r}}_{1},\ldots ,{\tilde{r}}_{K^{☆}}$ and $\rho_{1}^{☆},\ldots ,\rho_{K^{☆}}^{☆}$ and $\rho_{\infty }^{m}$ between 0 and ${\tilde{\rho }}_{\infty }$ such that

(A2) $$\begin{array}{c} \sum_{l=1}^{K^{☆}}{\tilde{r}}_{l}\log{\tilde{r}}_{l}=\sum_{l=1}^{K^{☆}}\rho_{l}^{☆}\log\rho_{l}^{☆}+\sum_{l=1}^{K^{☆}}\left(1+\log r_{l}^{m}\right)\left({\tilde{r}}_{l}-\rho_{l}^{☆}\right)\;\left(l=1,\ldots ,K^{☆}\right), \end{array}$$

(A3) $$\begin{array}{c} \left(1-{\tilde{\rho }}_{\infty }\right)\log\left(1-{\tilde{\rho }}_{\infty }\right)=\left(1-\log\left(1-\rho_{\infty }^{m}\right)\right){\tilde{\rho }}_{\infty }.\; \end{array}$$

Also, from assumption (C3), if *N* is sufficiently large, $\log r_{l}^{m}$ and $\log(1-\rho_{\infty }^{m})$ are finite because $r_{l}^{m}$ and $\rho_{\infty }^{m}$ become arbitrarily close to $\rho_{l}^{☆}\left(>0\right)$ and 0, respectively. Similarly, there exist $\rho_{1}^{m},\ldots ,\rho_{K^{☆}}^{m}$ between ${\tilde{\rho }}_{1}^{☆},\ldots ,{\tilde{\rho }}_{K^{☆}}^{☆}$ and $\rho_{1}^{☆},\ldots ,\rho_{K^{☆}}^{☆}$ such that

(A4) $$\sum_{l=1}^{K^{☆}}{\tilde{\rho }}_{l}^{☆}\log{\tilde{\rho }}_{l}^{☆}=\sum_{l=1}^{K^{☆}}\rho_{l}^{☆}\log\rho_{l}^{☆}+\sum_{l=1}^{K^{☆}}\left(1+\log\rho_{l}^{m}\right)\left({\tilde{\rho }}_{l}^{☆}-\rho_{l}^{☆}\right)\;\left(l=1,\ldots ,K^{☆}\right).\;$$

Also, from the central limit theorem, ${\tilde{\rho }}_{l}^{☆}$ converges to $\rho_{k}^{☆}$ at the speed of $O_{P}\left(1/\sqrt{N}\right)$.

Using (A2)–(A4), we can calculate as

$$\begin{array}{cc} \; & \;-\left(1-{\tilde{\rho }}_{\infty }\right)\sum_{l=1}^{K^{☆}}\frac{{\tilde{r}}_{l}}{1-{\tilde{\rho }}_{\infty }}\log\left(\frac{{\tilde{r}}_{l}}{1-{\tilde{\rho }}_{\infty }}\right)\\ = & \;-\sum_{l=1}^{K^{☆}}{\tilde{r}}_{l}\log{\tilde{r}}_{l}+K^{☆}\left(1-{\tilde{\rho }}_{\infty }\right)\log\left(1-{\tilde{\rho }}_{\infty }\right)\\ = & \;-\sum_{l=1}^{K^{☆}}\rho_{l}^{☆}\log\rho_{l}^{☆}+O_{P}\left(\sum_{l=1}^{K^{☆}}\left|{\tilde{r}}_{l}-\rho_{l}^{☆}\right|+{\tilde{\rho }}_{\infty }\right)\\ = & \;-\sum_{l=1}^{K^{☆}}{\tilde{\rho }}_{l}^{☆}\log{\tilde{\rho }}_{l}^{☆}+O_{P}\left(\sum_{l=1}^{K^{☆}}\left|{\tilde{r}}_{l}-\rho_{l}^{☆}\right|+{\tilde{\rho }}_{\infty }+\frac{1}{\sqrt{N}}\right). \end{array}$$

Next, we show that

$$\begin{array}{cc} \; & \;\frac{1}{N}\sum_{n=1}^{N}\sum_{l=1}^{K^{☆}}\left(\sum_{k\in I_{0}}\gamma_{k}\left(x_{n}\right)\right)\frac{r_{l}h_{l}\left(x_{n}\right)}{\sum_{l^{\prime }=1}^{K^{☆}}r_{l^{\prime }}h_{l^{\prime }}\left(x_{n}\right)}\log\left(\frac{r_{l}h_{l}\left(x_{n}\right)}{\sum_{l^{\prime }=1}^{K^{☆}}r_{l^{\prime }}h_{l^{\prime }}\left(x_{n}\right)}\right)\\ = & \;\frac{1}{N}\sum_{n=1}^{N}\sum_{l=1}^{K^{☆}}\gamma_{l}^{☆}\left(x_{n}\right)\log\gamma_{l}^{☆}\left(x_{n}\right)+O_{P}\left(∥\phi -\phi^{☆}∥+{\tilde{\rho }}_{\infty }\right). \end{array}$$

We first define the following functions for $l=1,\ldots ,K^{☆}$:

$$F_{l}\left(\phi ,\psi ,x\right):=\frac{r_{l}h_{l}\left(x\right)}{\sum_{l^{\prime }=1}^{K^{☆}}r_{l^{\prime }}h_{l^{\prime }}\left(x\right)}\log\left(\frac{r_{l}h_{l}\left(x\right)}{\sum_{l^{\prime }=1}^{K^{☆}}r_{l^{\prime }}h_{l^{\prime }}\left(x\right)}\right).$$

When $\phi =\phi^{☆}$, $F_{l}\left(\phi^{☆},\psi ,x\right)$ is calculated as $\gamma_{l}^{☆}\left(x\right)\log\gamma_{l}^{☆}\left(x\right)$; it is independent of ψ. In this case, we omit ψ and write $F_{l}\left(\phi^{☆},\psi ,x\right)$ as $F_{l}\left(\phi^{☆},x\right)$. Applying the mean-value theorem to this function, there exists a function 0<τ(x)<1 such that

(A5) $$F_{l}\left(\phi ,\psi ,x\right)=F_{l}\left(\phi^{☆},x\right)+{\left(\frac{\partial F_{l}\left(\phi^{☆}+t\left(x\right)\left(\phi -\phi^{☆}\right),\psi ,x\right)}{\partial \phi }\right)}^{⊤}\left(\phi -\phi^{☆}\right).\;$$

Moreover, it can be shown that $\partial F_{l}\left(\phi^{☆}+t\left(x\right)\left(\phi -\phi^{☆}\right),\psi ,x\right)/\partial \phi =O_{P}\left(1\right)$ uniformly with x,τ(x), and ψ. Indeed, letting

$$\begin{array}{c} \phi^{m}:=\left({\left\{\theta_{k}^{m}\right\}}_{k=1}^{i_{K^{☆}}},{\left\{r_{l}^{m}\right\}}_{l=1}^{K^{☆}},{\left\{\rho_{k}^{m}\right\}}_{k=i_{K^{☆}}+1}^{K}\right):=\phi^{☆}+t\left(x\right)\left(\phi -\phi^{☆}\right),\\ h_{l}^{m}\left(x\right):=\sum_{k\in I_{l}}s_{k}g_{k}\left(x|\theta_{k}^{m}\right),\\ \gamma_{l}^{m}\left(x\right):=\frac{r_{l}^{m}h_{l}^{m}\left(x\right)}{\sum_{l^{\prime }=1}^{K^{☆}}r_{l^{\prime }}^{m}h_{l^{\prime }}^{m}\left(x\right)},\; \end{array}$$

derivative of each parameter in ϕ is bounded as follows:

$$\begin{array}{cc} \; & \;\left|\frac{\partial F_{l}\left(\phi^{m},\psi ,x\right)}{\partial r_{l}}\right|\\ = & \;\left|\left(\frac{h_{l}^{m}\left(x\right)}{\sum_{l^{\prime }=1}^{K^{☆}}r_{l^{\prime }}^{m}h_{l^{\prime }}^{m}\left(x\right)}-\frac{r_{l}^{m}{\left(h_{l}^{m}\left(x\right)\right)}^{2}}{{\left(\sum_{l^{\prime }=1}^{K^{☆}}r_{l^{\prime }}^{m}h_{l^{\prime }}^{m}\left(x\right)\right)}^{2}}\right)\left(1+\log\left(\frac{r_{l}^{m}h_{l}^{m}\left(x\right)}{\sum_{l^{\prime }=1}^{K^{☆}}r_{l^{\prime }}^{m}h_{l^{\prime }}^{m}\left(x\right)}\right)\right)\right|\\ \leq & \;\frac{\gamma_{l}^{m}\left(x\right)}{r_{l}^{m}}+\frac{{\left(\gamma_{l}^{m}\left(x\right)\right)}^{2}}{r_{l}^{m}}+\frac{1}{r_{l}^{m}}\left|\gamma_{l}^{m}\left(x\right)\log\gamma_{l}^{m}\left(x\right)\right|+\frac{1}{r_{l}^{m}}\left|{\left(\gamma_{l}^{m}\left(x\right)\right)}^{2}\log\gamma_{l}^{m}\left(x\right)\right|\\ \leq & \;\frac{4}{r_{l}^{m}},\\ \; & \;\left|\frac{\partial F_{l}\;\left(\phi^{m},\psi ,x\right)}{\partial r_{m}}\right|\;\left(m\neq l\right)\\ = & \;\left|\frac{r_{l}^{m}h_{l}^{m}\left(x\right)h_{m}^{m}\left(x\right)}{{\left(\sum_{l^{\prime }=1}^{K^{☆}}r_{l^{\prime }}^{m}h_{l^{\prime }}^{m}\left(x\right)\right)}^{2}}\left(1+\log\left(\frac{r_{l}^{m}h_{l}^{m}\left(x\right)}{\sum_{l^{\prime }=1}^{K^{☆}}r_{l^{\prime }}^{m}h_{l^{\prime }}^{m}\left(x\right)}\right)\right)\right|\\ \leq & \;\frac{\gamma_{m}^{m}\left(x\right)}{r_{m}^{m}}\left|\gamma_{l}^{m}\left(x\right)\log\gamma_{l}^{m}\left(x\right)\right|\\ \leq & \;\frac{1}{r_{m}^{m}},\\ \; & \;∥\frac{\partial F_{l}\left(\phi^{m},\psi ,x\right)}{\partial \theta_{k}}∥\;\left(k\in I_{l}\right)\\ = & \;∥\left(\frac{r_{l}^{m}}{\sum_{l^{\prime }=1}^{K^{☆}}r_{l^{\prime }}^{m}h_{l^{\prime }}^{m}\left(x\right)}\frac{\partial h_{l}^{m}\left(x\right)}{\partial \theta_{k}}-\frac{{\left(r_{l}^{m}\right)}^{2}h_{l}^{m}\left(x\right)}{{\left(\sum_{l^{\prime }=1}^{K^{☆}}r_{l^{\prime }}^{m}h_{l^{\prime }}^{m}\left(x\right)\right)}^{2}}\frac{\partial h_{l}^{m}\left(x\right)}{\partial \theta_{k}}\right)\\ \; & \;\;\times \left(1+\log\left(\frac{r_{l}^{m}h_{l}^{m}\left(x\right)}{\sum_{l^{\prime }=1}^{K^{☆}}r_{l^{\prime }}^{m}h_{l^{\prime }}^{m}\left(x\right)}\right)\right)∥\\ \leq & \;\left(\gamma_{l}^{m}\left(x\right)+{\left(\gamma_{l}^{m}\left(x\right)\right)}^{2}+\left|\gamma_{l}^{m}\left(x\right)\log\gamma_{l}^{m}\left(x\right)\right|+\left|{\left(\gamma_{l}^{m}\left(x\right)\right)}^{2}\log\gamma_{l}^{m}\left(x\right)\right|\right)∥\frac{1}{h_{l}^{m}\left(x\right)}\frac{\partial h_{l}^{m}\left(x\right)}{\partial \theta_{k}}∥\\ \leq & \;4∥\underset{\theta \in \Theta_{\epsilon }}{\sup}\left(\frac{1}{g(x|\theta )}\frac{\partial g(x|\theta )}{\partial \theta_{k}}\right)∥,\\ \; & \;∥\frac{\partial F_{l}\left(\phi^{m},\psi ,x\right)}{\partial \theta_{k}}∥\;\left(k\in I_{m},m\neq l\right)\\ = & \;∥\frac{r_{l}^{m}r_{m}^{m}h_{l}^{m}\left(x\right)}{{\left(\sum_{l^{\prime }=1}^{K^{☆}}r_{l^{\prime }}^{m}h_{l^{\prime }}^{m}\left(x\right)\right)}^{2}}\frac{\partial h_{m}^{m}\left(x\right)}{\partial \theta_{k}}\left(1+\log\left(\frac{r_{l}^{m}h_{l}^{m}\left(x\right)}{\sum_{l^{\prime }=1}^{K^{☆}}r_{l^{\prime }}^{m}h_{l^{\prime }}^{m}\left(x\right)}\right)\right)∥\\ \leq & \;\left(\gamma_{m}^{m}\left(x\right)\gamma_{l}^{m}\left(x\right)+\gamma_{m}^{m}\left(x\right)\left|\gamma_{l}^{m}\left(x\right)\log\gamma_{l}^{m}\left(x\right)\right|\right)∥\frac{\partial h_{m}^{m}\left(x\right)}{\partial \theta_{k}}∥\\ \leq & \;2∥\underset{\theta \in \Theta_{\epsilon }}{\sup}\left(\frac{1}{g(x|\theta )}\frac{\partial g(x|\theta )}{\partial \theta_{k}}\right)∥,\\ \; & \;\left|\frac{\partial F_{l}\;\left(\phi^{m},\psi ,x\right)}{\partial \rho_{k}}\right|=0\;\left(k\in I_{\infty }\right), \end{array}$$

where

$$\Theta_{\epsilon }\left\{\theta \;|{\;}^{\exists }l\in \left\{1,\ldots ,K^{☆}\right\},∥\theta -\theta_{l}^{☆}∥<∥\phi -\phi^{☆}∥\right\}.$$

They are all finite because $r_{l}^{m}$ become arbitrarily close to $\rho_{l}^{☆}$ and $\Theta_{\epsilon }$ become arbitrarily smaller as N→∞; condition (C1) is also employed.

Using (A5), we can calculate as

$$\begin{array}{cc} \; & \;\frac{1}{N}\sum_{n=1}^{N}\sum_{l=1}^{K^{☆}}\left(\sum_{k\in I_{l}}\gamma_{k}\left(x_{n}\right)\right)\frac{r_{l}h_{l}\left(x_{n}\right)}{\sum_{l^{\prime }=1}^{K^{☆}}r_{l^{\prime }}h_{l^{\prime }}\left(x_{n}\right)}\log\left(\frac{r_{l}h_{l}\left(x_{n}\right)}{\sum_{l^{\prime }=1}^{K^{☆}}r_{l^{\prime }}h_{l^{\prime }}\left(x_{n}\right)}\right)\\ = & \;\frac{1}{N}\sum_{n=1}^{N}\sum_{l=1}^{K^{☆}}\left(1-\sum_{k\in I_{\infty }}\gamma_{k}\left(x_{n}\right)\right)\left(F_{l}\left(\phi^{☆},x_{n}\right)+{\left(\frac{\partial F_{l}\left(\phi^{m},\psi ,x_{n}\right)}{\partial \phi }\right)}^{⊤}\left(\phi -\phi^{☆}\right)\right)\\ = & \;\frac{1}{N}\sum_{n=1}^{N}\sum_{l=1}^{K^{☆}}\left(1-\sum_{k\in I_{\infty }}\gamma_{k}\left(x_{n}\right)\right)\left(\gamma_{l}^{☆}\left(x\right)\log\gamma_{l}^{☆}\left(x\right)+{\left(\frac{\partial F_{l}\left(\phi^{m},\psi ,x_{n}\right)}{\partial \phi }\right)}^{⊤}\left(\phi -\phi^{☆}\right)\right). \end{array}$$

Therefore,

$$\begin{array}{l} \begin{array}{c} \left|\frac{1}{N}\sum_{n=1}^{N}\sum_{l=1}^{K^{☆}}\left(\sum_{k\in I_{0}}\gamma_{k}\left(x_{n}\right)\right)\frac{r_{l}h_{l}\left(x_{n}\right)}{\sum_{l^{\prime }=1}^{K^{☆}}r_{l^{\prime }}h_{l^{\prime }}\left(x_{n}\right)}\log\left(\frac{r_{l}h_{l}\left(x_{n}\right)}{\sum_{l^{\prime }=1}^{K^{☆}}r_{l^{\prime }}h_{l^{\prime }}\left(x_{n}\right)}\right)\right.\\ \left.-\frac{1}{N}\sum_{n=1}^{N}\sum_{l=1}^{K^{☆}}\gamma_{l}^{☆}\left(x_{n}\right)\log\gamma_{l}^{☆}\left(x_{n}\right)\right| \end{array}\\ \leq \frac{1}{N}\sum_{n=1}^{N}\sum_{l=1}^{K^{☆}}\left|{\left(\frac{\partial F_{l}\left(\phi^{m},\psi ,x_{n}\right)}{\partial \phi }\right)}^{⊤}\left(\phi -\phi^{☆}\right)\right|\\ \;\;+\frac{1}{N}\sum_{n=1}^{N}\sum_{l=1}^{K^{☆}}\;\left(\sum_{k\in I_{\infty }}\gamma_{k}\left(x_{n}\right)\right)\left|\gamma_{l}^{☆}\left(x_{n}\right)\log\gamma_{l}^{☆}\left(x_{n}\right)\right|\\ \leq \;\sum_{l=1}^{K^{☆}}\underset{x_{n},\psi }{\sup}\left(∥\frac{\partial F_{l}\left(\phi^{m},\psi ,x_{n}\right)}{\partial \phi }∥∥\phi -\phi^{☆}∥\right)+3K^{☆}{\tilde{\rho }}_{\infty }\\ =\;O_{P}\left(∥\phi -\phi^{☆}∥+{\tilde{\rho }}_{\infty }\right). \end{array}$$

Step 4

Finally, we show that

$$\sum_{l=1}^{K^{☆}}\left(\sum_{k\in I_{l}}{\tilde{\rho }}_{k}\right)\mathrm{MC}\left({\left\{\frac{s_{k}g_{k}\left(x_{n}\right)}{h_{l}\left(x_{n}\right)}\right\}}_{k\in I_{l}};\left\{\sum_{k\in I_{l}}\gamma_{k}\left(x_{n}\right)\right\}\right)=O_{P}\left(∥\phi -\phi^{☆}∥\right)$$

for every $l=1,\ldots ,K^{☆}$.

To this end, we write the left hand as $G({\left\{\theta_{k}\right\}}_{k\in I_{l}})$ and consider it as a function of ${\left\{\theta_{k}\right\}}_{k\in I_{l}}$. Then, for all other parameters, $G({\left\{\theta_{k}^{☆}\right\}}_{k\in I_{l}})=0$. Also, the derivative of G by $\theta_{l}$ is $O_{P}\left(1\right)$ as N→∞. Indeed, it can be rewritten as

$$\begin{array}{cc} \; & \;G\left({\left\{\theta_{k}\right\}}_{k\in I_{l}}\right)\\ = & \;\frac{1}{N}\sum_{n=1}^{N}\sum_{k\in I_{l}}\frac{r_{l}s_{k}g_{k}\left(x_{n}\right)}{f\left(x_{n}\right)}\log\left(\frac{s_{k}g_{k}\left(x_{n}\right)}{h_{l}\left(x_{n}\right)}\right)\\ \; & \;\;-\frac{1}{N}\sum_{k\in I_{l}}\left(\sum_{n=1}^{N}\frac{r_{l}s_{k}g_{k}\left(x_{n}\right)}{f\left(x_{n}\right)}\right)\log\left(\sum_{n^{\prime }=1}^{N}\frac{r_{l}s_{k}g_{k}\left(x_{n^{\prime }}\right)}{f\left(x_{n^{\prime }}\right)}\right)\\ \; & \;\;+\frac{1}{N}\sum_{k\in I_{l}}\left(\sum_{n=1}^{N}\frac{r_{l}s_{k}g_{k}\left(x_{n}\right)}{f\left(x_{n}\right)}\right)\log\left(\sum_{n^{\prime }=1}^{N}\frac{r_{l}h_{l}\left(x_{n^{\prime }}\right)}{f\left(x_{n^{\prime }}\right)}\right). \end{array}$$

Also, we define the posterior probabilities within $h_{l}$ as

$$\gamma_{k}^{\left(l\right)}\left(x\right)\frac{s_{k}g_{k}\left(x\right)}{h_{l}\left(x\right)}\;\left(k\in I_{l}\right).$$

Then, the derivatives are bounded as

$$\begin{array}{cc} \; & \;∥\frac{\partial G\left({\left\{\theta_{k}\right\}}_{k\in I_{l}}\right)}{\partial \theta_{m}}∥\;\left(m\in I_{l}\right)\\ = & \;∥\frac{1}{N}\sum_{n=1}^{N}\sum_{k\in I_{l}}\left(\frac{r_{l}s_{k}}{f\left(x_{n}\right)}\frac{\partial g_{k}\left(x_{n}\right)}{\partial \theta_{m}}-\frac{r_{l}^{2}s_{k}s_{m}g_{k}\left(x_{n}\right)}{{\left(f\left(x_{n}\right)\right)}^{2}}\frac{\partial g_{m}\left(x_{n}\right)}{\partial \theta_{m}}\right)\\ \; & \;\;\times \left(\log\left(\frac{s_{k}g_{k}\left(x_{n}\right)}{h_{l}\left(x_{n}\right)}\right)-\log\left(\sum_{n^{\prime }=1}^{N}\frac{r_{l}s_{k}g_{k}\left(x_{n^{\prime }}\right)}{f\left(x_{n^{\prime }}\right)}\right)+\log\left(\sum_{n^{\prime \prime }=1}^{N}\frac{r_{l}h_{l}\left(x_{n^{\prime \prime }}\right)}{f\left(x_{n^{\prime \prime }}\right)}\right)\right)\\ \; & \;\;+\frac{1}{N}\sum_{n=1}^{N}\sum_{k\in I_{l}}\left(\frac{r_{l}s_{k}}{f\left(x_{n}\right)}\frac{\partial g_{k}\left(x_{n}\right)}{\partial \theta_{m}}-\frac{r_{l}s_{k}s_{m}g_{k}\left(x_{n}\right)}{f\left(x_{n}\right)h_{l}\left(x_{n}\right)}\frac{\partial g_{m}\left(x_{n}\right)}{\partial \theta_{m}}\right)\\ \; & \;\;-\frac{1}{N}\sum_{k\in I_{l}}\sum_{n=1}^{N}\left(\frac{r_{l}s_{k}}{f\left(x_{n}\right)}\frac{\partial g_{k}\left(x_{n}\right)}{\partial \theta_{m}}-\frac{r_{l}^{2}s_{k}s_{m}g_{k}\left(x_{n}\right)}{{\left(f\left(x_{n}\right)\right)}^{2}}\frac{\partial g_{m}\left(x_{n}\right)}{\partial \theta_{m}}\right)\\ \; & \;\;+\frac{1}{N}\sum_{k\in I_{l}}\frac{\sum_{n^{\prime }=1}^{N}\frac{r_{l}s_{k}g_{k}\left(x_{n^{\prime }}\right)}{f\left(x_{n^{\prime }}\right)}}{\sum_{n^{\prime \prime }=1}^{N}\frac{h_{l}\left(x_{n^{\prime \prime }}\right)}{f\left(x_{n^{\prime \prime }}\right)}}\sum_{n=1}^{N}\left(\frac{r_{l}s_{m}}{f\left(x_{n}\right)}\frac{\partial g_{k}\left(x_{n}\right)}{\partial \theta_{m}}-\frac{r_{l}s_{m}h\left(x_{n}\right)}{{\left(f\left(x_{n}\right)\right)}^{2}}\frac{\partial g_{m}\left(x_{n}\right)}{\partial \theta_{m}}\right)∥\\ \leq & \;\frac{1}{N}\sum_{n=1}^{N}\sum_{k\in I_{l}}\left|\gamma_{k}^{\left(l\right)}\left(x_{n}\right)\log\gamma_{k}^{\left(l\right)}\left(x_{n}\right)\right|∥\frac{1}{g_{k}\left(x_{n}\right)}\frac{\partial g_{k}\left(x_{n}\right)}{\partial \theta_{m}}∥\\ \; & \;\;+\frac{1}{N}\sum_{n=1}^{N}\sum_{k\in I_{l}}\left|\gamma_{k}^{\left(l\right)}\left(x_{n}\right)\gamma_{m}^{\left(l\right)}\left(x_{n}\right)\log\gamma_{k}^{\left(l\right)}\left(x_{n}\right)\right|∥\frac{1}{g_{m}\left(x_{n}\right)}\frac{\partial g_{m}\left(x_{n}\right)}{\partial \theta_{m}}∥\\ \; & \;\;+\sum_{k\in I_{l}}\left[\frac{2}{N}\sum_{n=1}^{N}\frac{r_{l}h_{l}\left(x_{n}\right)}{f\left(x_{n}\right)}\underset{\theta \in \Theta_{\epsilon }}{\sup}∥\frac{1}{g\left(x_{n}|\theta \right)}\frac{\partial g\left(x_{n}|\theta \right)}{\partial \theta }∥\right.\\ \; & \;\;\times \left.\left|\frac{\sum_{n^{\prime }=1}^{N}\frac{r_{l}s_{k}g_{k}\left(x_{n^{\prime }}\right)}{f\left(x_{n^{\prime }}\right)}}{\sum_{n^{\prime }=1}^{N}\frac{r_{l}h_{l}\left(x_{n^{\prime }}\right)}{f\left(x_{n^{\prime }}\right)}}\log\left(\frac{\sum_{n^{\prime }=1}^{N}\frac{r_{l}s_{k}g_{k}\left(x_{n^{\prime }}\right)}{f\left(x_{n^{\prime }}\right)}}{\sum_{n^{\prime }=1}^{N}\frac{r_{l}h_{l}\left(x_{n^{\prime }}\right)}{f\left(x_{n^{\prime }}\right)}}\right)\right|\right]\\ \; & \;\;+\frac{2}{N}\sum_{n=1}^{N}\sum_{k\in I_{l}}\left|\gamma_{k}^{\left(l\right)}\left(x_{n}\right)\right|∥\frac{1}{g_{k}\left(x_{n}\right)}\frac{\partial g_{k}\left(x_{n}\right)}{\partial \theta_{m}}∥\\ \; & \;\;+\frac{2}{N}\sum_{n=1}^{N}\sum_{k\in I_{l}}\left|\gamma_{k}^{\left(l\right)}\left(x_{n}\right)\gamma_{m}^{\left(l\right)}\left(x_{n}\right)\right|∥\frac{1}{g_{m}\left(x_{n}\right)}\frac{\partial g_{m}\left(x_{n}\right)}{\partial \theta_{m}}∥\\ \leq & \;8K\underset{\theta \in \Theta_{\epsilon }}{\sup}∥\frac{1}{g\left(x_{n}|\theta \right)}\frac{\partial g\left(x_{n}|\theta \right)}{\partial \theta }∥\\ = & \;O_{P}\left(1\right), \end{array}$$

where, it is assumed that ${\left\{\theta_{k}\right\}}_{k\in I_{l}}$ are sufficiently close to $\theta_{k}^{☆}$, which holds if *N* is sufficiently large because of condition (C2).

Therefore, by the mean-value theorem, there exist ${\left\{\theta_{k}^{m}\right\}}_{k\in I_{l}}$ such that

$$\begin{array}{cc} G({\left\{\theta_{k}\right\}}_{k\in I_{l}})\; & =\sum_{k\in I_{l}}\;{\left(\frac{\partial G\left({\left\{\theta_{k}^{m}\right\}}_{k\in I_{l}}\right)}{\partial \theta_{k}}\right)}^{⊤}\left(\theta_{k}^{m}-\theta_{k}^{☆}\right)\\ \; & =O_{P}\left(∥\phi -\phi^{☆}∥\right), \end{array}$$

which concludes the proof.
